# Phosphoproteomics identify arachidonic-acid-regulated signal transduction pathways modulating macrophage functions with implications for ovarian cancer

**DOI:** 10.7150/thno.52442

**Published:** 2021-01-01

**Authors:** Raimund Dietze, Mohamad K. Hammoud, María Gómez-Serrano, Annika Unger, Tim Bieringer, Florian Finkernagel, Anna M. Sokol, Andrea Nist, Thorsten Stiewe, Silke Reinartz, Viviane Ponath, Christian Preußer, Elke Pogge von Strandmann, Sabine Müller-Brüsselbach, Johannes Graumann, Rolf Müller

**Affiliations:** 1 Tranlational Oncology Group, Center for Tumor Biology and Immunology, Philipps University, Marburg, Germany.; 2 Biomolecular Mass Spectrometry, Max-Planck-Institute for Heart and Lung Research, Bad Nauheim, Germany.; 3 The German Centre for Cardiovascular Research (DZHK), Partner Site Rhine-Main, Max Planck Institute for Heart and Lung Research, Bad Nauheim, Germany.; 4 Genomics Core Facility, Philipps University, Marburg, Germany.; 5 Institute for Tumor Immunology, Philipps University, Marburg, Germany.; 6 Present address: Hochschule Landshut, 84036 Landshut, Germany.

**Keywords:** arachidonic acid, phosphoproteomics, macropinocytosis, ovarian cancer

## Abstract

Arachidonic acid (AA) is a polyunsaturated fatty acid present at high concentrations in the ovarian cancer (OC) microenvironment and associated with a poor clinical outcome. In the present study, we have unraveled a potential link between AA and macrophage functions.

**Methods:** AA-triggered signal transduction was studied in primary monocyte-derived macrophages (MDMs) by phosphoproteomics, transcriptional profiling, measurement of intracellular Ca^2+^ accumulation and reactive oxygen species production in conjunction with bioinformatic analyses. Functional effects were investigated by actin filament staining, quantification of macropinocytosis and analysis of extracellular vesicle release.

**Results:** We identified the ASK1 - p38δ/α (MAPK13/14) axis as a central constituent of signal transduction pathways triggered by non-metabolized AA. This pathway was induced by the Ca^2+^-triggered activation of calmodulin kinase II, and to a minor extent by ROS generation in a subset of donors. Activated p38 in turn was linked to a transcriptional stress response associated with a poor relapse-free survival. Consistent with the phosphorylation of the p38 substrate HSP27 and the (de)phosphorylation of multiple regulators of Rho family GTPases, AA impaired actin filament organization and inhibited actin-driven macropinocytosis. AA also affected the phosphorylation of proteins regulating vesicle biogenesis, and consistently, AA enhanced the release of tetraspanin-containing exosome-like vesicles. Finally, we identified phospholipase A_2_ group 2A (PLA2G2A) as the clinically most relevant enzyme producing extracellular AA, providing further potentially theranostic options.

**Conclusion:** Our results suggest that AA contributes to an unfavorable clinical outcome of OC by impacting the phenotype of tumor-associated macrophages. Besides critical AA-regulated signal transduction proteins identified in the present study, PLA2G2A might represent a potential prognostic tool and therapeutic target to interfere with OC progression.

## Introduction

Cancer progression, metastatic spread and escape of tumor cells from immune surveillance are crucially dependent on the signaling network of the tumor microenvironment (TME) 1. In ovarian cancer (OC), the peritoneal fluid (or ascites at advanced stages) is a crucial component of the peritoneal TME 2, 3. Ascites contains large numbers of tumor spheroids and immune cells, in particular tumor-associated macrophages (TAMs), T cells and NK cells, which produce soluble factors and extracellular vesicles 4-6, collectively referred to as the tumor secretome. In spite of its key role in OC progression and therapy resistance, many crucial components of this secretome have not been identified, and the cellular origins and targets of individual mediators as well as their biological functions in the crosstalk between tumor and host cells remain obscure.

Cytokines and growth factors released into the TME are pivotal to all aspects of tumor growth, progression, dissemination and immune escape by affecting numerous biological processes, including cell proliferation, differentiation, survival and motility, immune cell attraction and activation as well as angiogenesis 1, 3. Another essential class of soluble mediators with similar functions in the TME are bioactive lipids 7, 8. These include breakdown products of phospholipids, i.e., lysophosphatidic acids (LPAs), polyunsaturated fatty acids (PUFAs) and their metabolites, in particular those derived from arachidonic acid (AA) by the cyclooxygenase and lipoxygenase pathways. The importance of lipid mediators for tumorigenesis is probably best understood for LPA in cancer cell invasion 9-11, and for prostaglandin E_2_ in immune suppression and angiogenesis 12. Intriguingly, the ascites levels of several bioactive lipids, including several LPA species, AA and AA-derived eicosanoids (with median AA levels as high as 35 µM) are associated with a short relapse-free survival (RFS) of OC 13, 14. As the ascites levels of these mediators do not correlate with one another, they are most likely independently associated with RFS.

While the pro-tumorigenic functions of LPA and AA metabolites are partly understood, it is unclear whether, and if so how, non-metabolized AA might be able to exert tumorigenesis-promoting functions. AA can interact with cellular proteins in various ways, which include the membrane-bound G-protein-coupled receptor (GPCR) free fatty acid receptor 4 (FFAR4) 15-17 and the nuclear receptor PPARβ/δ 18. PPARβ/δ is indeed activated in TAMs by PUFAs present in OC ascites at high concentrations 19. It is, however, unlikely that PPARβ/δ mediates the adverse effect of AA on RFS, because the major ascites-associated PUFA linoleic acid with strong agonistic effect on PPARβ/δ is not linked to survival 19.

AA has also been linked to the production of reactive oxygen species (ROS), partly via NADPH oxidase NOX-2 located in the plasma membrane 20-22, but the functional relevance of this mechanism remains unclear. Other targets of non-metabolized AA have been identified, including protein kinase C 23-27 and the MAP kinases p38 and JNK 28-30. In monocytes and neutrophils, the AA-triggered induction of p38 phosphorylation was dependent on Ca^2+^, phospholipase C, and/or a pertussis toxin (PTX)-sensitive signaling compound, indicative of signaling via a GPCR 28, 29. Whether FFAR4 is involved in this AA-regulated pathway is currently unknown. A role for GPCR(s) in AA-induced signaling is also suggested by the observation that AA-triggered superoxide generation 22 and extracellular signal-regulated kinase (ERK) activation are inhibited by PTX 31. It remains, however, unclear how these events are interconnected and contribute to signaling transduction cascades, transcriptional programs and cancer-related biological functions. Non-metabolized AA can also exert direct effects on cellular membranes, leading to in altered mechanical properties of the bilayer, which can modulate the function of membrane channels 21 and transmembrane receptors 32.

TAMs represent one of the most prominent host cell types in ascites and promote tumor growth, metastasis, immunosuppression and chemoresistance. The pivotal role of TAMs in the TME has not only been shown in mouse models but is also evident in cancer patients, where the intratumoral macrophage density is associated with a poor clinical outcome 33, which also applies to OC 34. These findings are consistent with the shortened RFS observed in OC patients with a high frequency of CD163^high^ TAMs in ascites 35.

TAMs can be derived from both resident tissue macrophages and blood monocytes 36. They are polarized by factors of the TME to adopt mixed-polarized phenotypes with components of both inflammatory and alternatively activated macrophages 35-39, but the underlying signaling mechanisms are only partly understood. In the present study, we have addressed the question whether AA modulates signal transduction pathways in macrophages and thereby interferes with their physiological functions. To this end we used monocyte-derived macrophages (MDMs) as an experimental system to mimic the infiltration of monocytes/macrophages into the tumor microenvironment with is high levels of AA.

## Materials and Methods

### Isolation and culture of monocyte-derived macrophages (MDMs) from healthy donors

Leukoreduction System (LRS) chambers with leucocytes from healthy adult volunteers were kindly provided by the Center for Transfusion Medicine and Hemotherapy at the University Hospital Gießen and Marburg. Mononuclear cells were isolated by Ficoll density gradient centrifugation. Subsequently, monocytes were purified by adherence selection and used for subsequent differentiation in RPMI1640 (Life Technologies, Darmstadt, Germany) supplemented with 5 % human AB serum (Sigma), 1 mM sodium pyruvate (Sigma Aldrich, Taufkirchen, Germany) at approximately 5 × 10^7^ cells per 150 mm dish, 1.5 × 10^7^ cells per 100 mm dish or 2.5 × 10^6^, 1 × 10^6^, 0.5 × 10^6^ or 0.1 × 10^6^ cells per well in 6-well, 12-well, 24-well or 96-well plates, respectively. The adherent cells were allowed to differentiate for at least 7 days and used within 3 days thereafter. Under these culture conditions, the macrophage-specific markers CD206 (MRC1) and HLA-DR were >95% as determined by flow cytometry. For the last 24 h prior to any experiment, the medium was replaced with serum-free medium for serum starvation.

### Small-molecule compounds

Fatty acids, Latrunculin A, AH7614, BAPTA-AM and U73122 were obtained from Cayman chemicals (Hamburg, Germany), YM-254890 and BIRB796 (Doramapimod) from Biomol (Hamburg, Germany), pertussis toxin from Merck (Darmstadt, Germany), and SB203580 and Selonsertib from Biozol (Eching, Germany).

### RNA Sequencing

MDMs were treated with 50 µm AA or solvent (ethanol) for 3 h and total RNA was isolated using the NucleoSpin RNA II kit (740955.250, Macherey-Nagel, Düren, Germany). RNA-Seq was performed and data were processed as described previously (Reinartz et al., 2016; Worzfeld et al., 2018) using Ensembl 96 40. RNA-Seq data were deposited at EBI ArrayExpress (accession numbers E-MTAB-8833). Another RNA-Seq dataset for MDMs and TAM was used for Fig. 3E and Table S7 19, 41 (accession numbers E-MTAB-3114, E-MTAB-3398 and E-MTAB-3167). These datasets are not directly comparable because cDNA libraries were constructed by different methods (Illumina Truseq mRNA kit v2 for E-MTAB-8833, Epicentre Scriptseq human/mouse/rat low input Gold for E-MTAB-3114, E-MTAB-3398 and E-MTAB-3167). RNA expression data for solid tumor tissue from OC patients was retrieved from The Cancer Genome Atlas (TCGA) (https://www.cancer.gov/tcga).

### Proteomic and phosphoproteomics analyses

Differentiation of the cells was performed as described above. The cells were serum starved for 40 h and then treated with 50 µM AA or solvent for 15 min. Proteomic and phosphoproteomic analyses were performed in triplicate as described 42 with modifications. 4% SDS, 100 mM Tris, pH 7.6 supplemented with PhosStop and complete protease inhibitor cocktail (both Roche) was used as a lysis buffer, followed by 10 min boiling and sonication prior to centrifugational extract clearing performed at 12000 rpm for 15 min at room temperature. Post-trypsinization, samples were acidified bringing them to 0.75% TFA and cleared by centrifugation. Starting from 400 μg protein extract per condition and replicate, 150 μg of peptides each were TMT labeled (6plex; channels 126-129) and combined by replicate to 600 μg samples, of which 50 μg were subjected to high pH reverse phase separation and 550 μg to TiSH 43. Following the batch enrichment steps of TiSH, ¾ of the product were HILIC fractionated and ¼ Measured unfractionated. Documentation of liquid chromatography/tandem mass spectrometry (LC/MS2) instrumentation and parametrization was extracted as well as summarized from raw data using MARMoSET 44 and is provided in the supplemental material (Table S1). The MaxQuant suite of algorithms 45, 46 (v. 1.6.8.0) together with the human canonical and isoforms UniProt database (downloaded on 2019/08/19; 173799 entries) and parametrized as included in the supplemental information (Table S2) were used for peptide/spectrum matching and quantitation. Phosphosite occupancies were normalized by dividing phosphosite intensities by the median protein group intensities associated with them using R. The mass spectrometry proteomics data have been deposited to the ProteomeXchange Consortium via the PRIDE partner repository 47 with the dataset identifier PXD021038 (http://proteomecentral.proteomexchange.org). *Reviewer PRIDE account details: Username 'reviewer33406@ebi.ac.uk', password 'IpfMUJwR'.*

### Immunoblotting and quantification

Immunoblots were performed according to standard Western blotting protocols using the following antibodies: pp38 T180/Y182 (4511, Cell Signaling, Frankfurt, Germany), pHSP27 S82 (SC-16669, Santa Cruz Biotechnology, Heidelberg, Germany), pCREB S133 (9198, Cell signaling), pCamKII T287 (PA-537833, Life Technologies Carlsbad, CA) p38 (9228, Cell Signaling), HSP27 (SC13132, Santa Cruz Biotechnology), CREB (9104, Cell Signaling), pan-CamKII (4436, Cell Signaling), β-Actin, (A5441,Sigma), α-rabbit IgG HRP-linked AB (27, Cell Signaling) and α-mouse IgG HRP-linked AB (32, Cell Signaling). Imaging and quantification were carried out using the ChemiDoc MP system and Image Lab software version 5 (Bio-Rad, Hercules, CA, USA). As phospho-p38 was frequently not detectable in solvent control samples, immunoblots were quantified relative to AA-treated samples for all immunoblots as indicated in the respective figure legends. In addition, the phosphoform signals were normalized against the respective protein signals to correct for unequal protein expression or loading.

Out of the four known CamKII isoforms, MDMs express mRNA for CamKIIβ, -γ and -δ, with CamKIIδ as the main form. The phosphoforms of CamKIIγ and -δ were readily detectable using the polyclonal phospho-CamKIIβ, -γ and -δ antibody (see above). The monoclonal pan-CamKII antibody used for CamKII detection, however, was unable to detect any CamKII isoform besides the δ variant, presumably because CamKIIδ is expressed at approximately 10-fold higher levels in MDMs compared to the other isoforms. For this reason, the upper pCaMKII band, representing pCamKIIγ, was normalized against β-actin.

### Reactive oxygen species (ROS) assay

MDMs were prepared and differentiated in 12-well plates as described above. A total ROS/superoxide detection kit from Enzo Life Sciences was used according to the manufacturer's protocol. In brief, macrophages were pretreated with the ROS scavenger N-acetylcysteine (NAC) or the NOX2 inhibitor VAS3947 for 60 min prior to exposure to AA, ETYA or solvent. Cells were treated with AA and stained simultaneously with ROS/superoxide detection reagent for 30 min. Pyocyanin-treated samples served as positive controls. After the incubation period the cells were washed once with assay buffer, gently scraped off the plates and ROS levels were analyzed by flow cytometry (FACSCanto II BD Biosciences). Gating strategies and compensations were performed according to the assay user manual.

### Measurement of intracellular free calcium

Calcium assays were carried out in 96-well microplate using a Fura-2 Calcium Flux Assay Kit (ab176766; abcam, Cambridge, UK) according to the instructions of the manufacturer with MDMs cultured as described above. Shortly, the MDMs were incubated with Fura-2 solution for 60 and 20 min at 37°C and room temperature, respectively. Kinetic measurements at 340 and 380 nm were carried out on a Victor Nivo Microplate Readers (PerkinElmer, Waltham, USA) for 0-105 sec after the addition of AA, ETYA or solvent to MDMs, and 340/380 nm excitation ratios were calculated. The 340/380 nm ratio before each treatment was used as reference for normalization.

### Staining of actin filaments

MDMs were differentiated on glass coverslips as described above. For actin filament staining the cells were incubated with 50 µM AA for 24 h, fixed with 4 % paraformaldehyde for 10 min at room temperature and permeabilized with 0.3% Triton X100 for 5 min. Actin filament was stained with 1:1000 diluted Phalloidin-California Red Conjugate (AAT Bioquest, Sunnyvale, USA) for 30 min at RT. Glass coverslips were mounted onto the microscope slides using a drop of mounting medium with DAPI (VEC-H-1200, Vector, Burlingame, USA) and sealed with nail polish. Images were taken at 100× magnification and processed on a widefield microscope (Leica DM5500, Leica Microsystems, Wetzlar, Germany).

### Macropinocytosis assay

MDMs cultured as described above were treated with AA 50 µM for 23 h, 200 nM of Latrunculin for 30 min A or solvent (ethanol) for 30 min. Macropinocytotic activity was evaluated the following day 60 minutes after addition of FITC-Dextran (70 kDa, 0.5 mg/ml). After gently scraping the cells of the culture plates, the samples were analyzed by flow cytometry. Gating was performed against cells preincubated for 60 min on ice to subtract FITC-Dextran binding from the uptake. Latrunculin is a powerful inhibitor of phagocytosis and macropinocytosis and was therefore used as an additional control.

### Isolation and characterization of EVs

After 7 days of differentiation, culture medium was replaced by fresh serum-free RPMI1640 supplemented with 1% sodium pyruvate, and AA at a final concentration of 50 µM or solvent (ethanol) control. Supernatants were harvested after 0 and 24 hrs of incubation from MDMs at a maximum cell density of 80%. Conditioned media were sequentially centrifuged (at 4°C) at 300 × g for 10 min to remove cell debris, 2,000 × g for 15 min to remove organelles, and 10,000 × g for 1 hr to remove large particles. EV release was determined in 10,000 × g supernatants (0.5 ml per 6-well) by high-sensitivity flow cytometry (HSFC). Time-point zero sample media were used as blanks. The presence of protein aggregates or other supramolecular complexes was estimated by treating 10,000 × g supernatants with Triton X-100 at a final concentration of 0.05% to disrupt EVs, as previously described 48. Further isolation of EVs was performed from culture supernatants (12 ml per 150 mm dish) by additional centrifugation at 100,000 × g for 90 min at 4°C in an Optima^TM^ XPN-80 ultracentrifuge equipped with an SW 41 Ti Swinging-bucket rotor (Beckman Coulter; Krefeld, Germany). After ultracentrifugation, EV pellets were re-suspended in 1 ml PBS and further centrifuged at 100,000 x g for 100 min in an Optima^TM^ MAX-XP ultracentrifuge (TLA-45 fixed-angle rotor, Beckman Coulter, Inc.). EV samples were re-suspended in 50 µl PBS and stored at -20°C until further analysis.

As recommended by the MISEV 2018 guidelines 49, three tetraspanin markers were assessed by HSFC in order to verify the EV nature of the samples. Immunostaining of at least 2 × 10^7^ particles in a final volume of 100 µl PBS was performed over-night at 4°C with FITC-coupled antibodies against human CD9 (200 ng, Cat. 312104), CD63 (100 ng, Cat. 353006) and CD81 (200 ng, Cat. 349504; all from BioLegend, Koblenz, Germany). Isotype control antibodies were used as negative controls (200 ng, BioLegend). Removing of unlabeled antibody was performed by PBS washing and subsequent ultracentrifugation at 100,000 × g for 100 min (4°C).

Particle concentration of EV samples was determined by HSFC and NanoFCM Professional Version (V1.0) software analysis. Immunostaining data from HSFC (FCS 3.0 files) were analyzed using FlowJo^TM^ (v10) software. Staining was considered positive when the percentage of positive events showed a minimum fold change of 3 relative to the matching isotype control.

### High-sensitivity flow cytometry (HSFC) fluorescence analysis of EVs

A Flow NanoAnalyzer fitted with a Blue Laser (488 nm) (NanoFCM, Inc.) was used for the multiparameter analysis of EV samples, allowing for the quantitative analysis of single particles down to 40 nm with high sensitivity. All experiments were performed in compliance with the NanoFCM system's recommendations (more information on http://www.nanofcm.com/). Briefly, dilutions of all samples were individually tested in order to record a total number of 3,000-12,500 events. PBS or RPMI1640 time-point zero samples were used for threshold setting and as blanks. Monodisperse silica nanoparticles cocktail (68-155 nm, Cat. S16M-Exo, NanoFCM, Inc.), and polystyrene 200 nm beads at a 1/100 dilution (QC Beads, Cat. S08210, NanoFCM, Inc.), were used as size and particle concentration reference, respectively. Standard beads and all samples were collected under the same sampling pressure (1 kPa) for 1 min. Light scattering was used for the measurement of nanoparticle size and size distributions. The EV size range was set between 40-300 nm. Fluorescence analyses were based on FITC-A. All applicable assay controls to HSFC described by MIFLowCyt-EV guidelines 50 (i.e. buffer controls, unstained samples or detergent treatment of stained samples) were evaluated. All samples were measured with at least two technical replicates.

### Statistical analysis

Comparative data were statistically analyzed by unpaired or paired Student's *t* test (two-sided, equal variance) as indicated. Significance levels are indicated as ****, ***, ** and * for *p <* 0.0001, *p <* 0.001, *p <* 0.01 and *p <* 0.05, respectively. Box pots in Fig. 3 and S1 depicting medians (line), upper and lower quartiles (box), range (whiskers) and outliers/fliers (diamonds) were constructed using the Seaborn boxplot function with Python. Variance of expression in Table 1 was determined by means of the Python function pandas.DataFrame.var(). Progression-free survival data for serous OC patients were obtained from the Kaplan-Meier Plotter meta-analysis database (https://kmplot.com; version 06/2020 with data for 2.190 OC patients) 51. Data associating gene expression with overall survival (OS) of OC were obtained from the PRECOG database (https://precog.stanford.edu) 52.

## Results

### Phosphoproteome analysis of AA-treated MDMs

**Figure 1:**
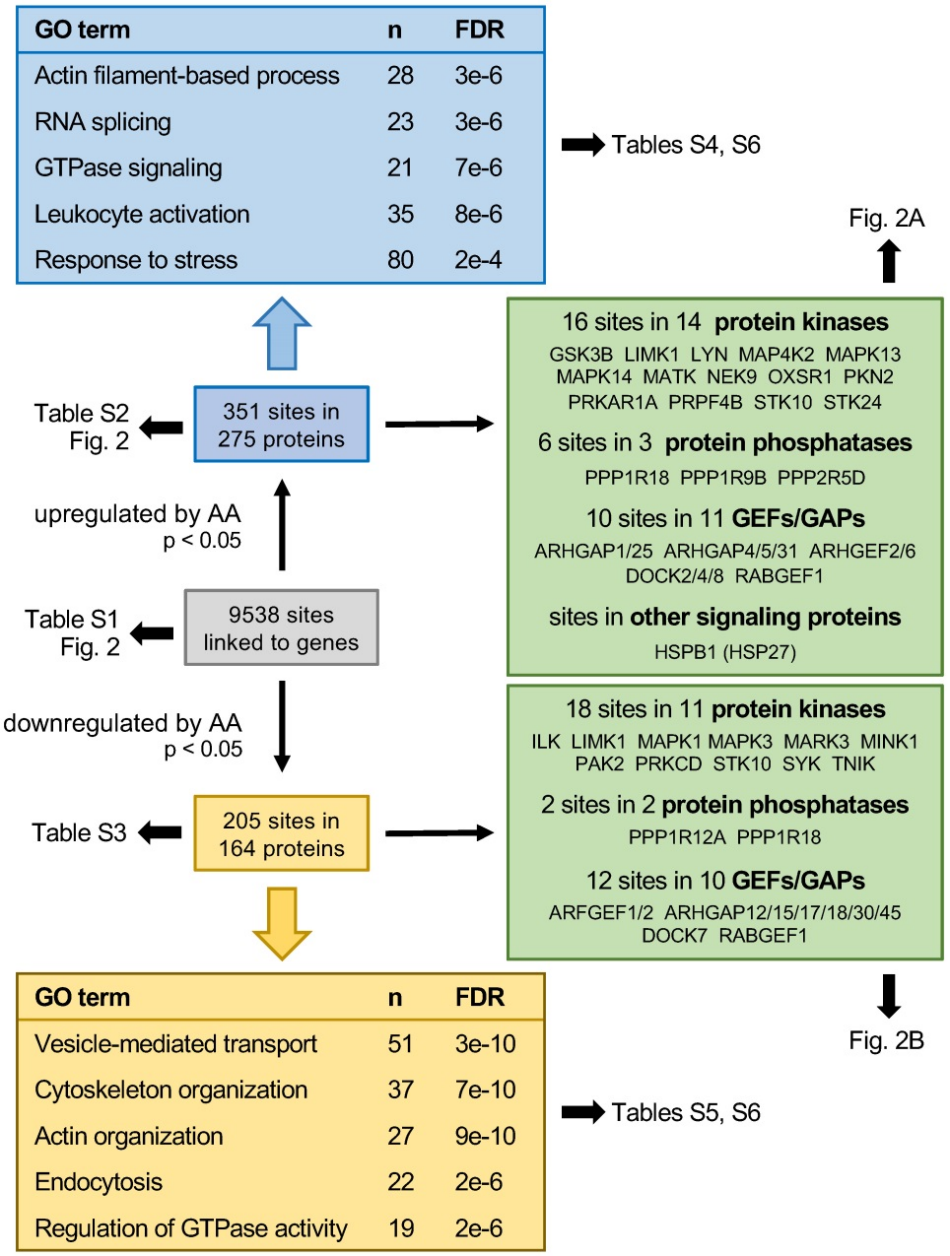
** Summary of the phosphoproteome analysis of AA-treated MDMs.** Blue boxes show the data for significantly upregulated phosphosites (nominal p value < 0.05; n = 3), yellow boxes the results downregulated phosphosites. Boxes at the top and bottom show for the most significantly (FDR) enriched terms associated with specific functions. n: number of proteins linked to the respective GO term. Green boxes: AA regulated phosphoproteins with functions in signal transduction mediated by protein kinases, phosphatases, G proteins (GTPases) or heat shock protein 27 (HSP27).

Mass-spectrometry-based phosphoproteomics identified 9538 phosphosites in MDMs treated with 50 µM AA or solvent for 15 min (see schematic summary in Fig. 1; Table S3; values normalized for total proteome signals). After normalization (see Materials and Methods), 351 of these sites sites in 275 proteins were significantly upregulated by AA (nominal *p* value < 0.05 by *t* test; Table S4), while 205 sites in 164 proteins were significantly downregulated (Table S5). These included 28 upregulated and 23 downregulated phosphosites, respectively, in signal-transducing protein kinases, phosphatases and G-protein regulators (Fig. 1). Gene ontology (GO) term enrichment analysis of upregulated phosphosites identified actin filament-based process, GTPase signaling, leukocyte activation and stress response as the most significant and enriched terms defining specific functions (Fig. 1; Table S6). The analogous analyses of downregulated phosphosites also yielded cytoskeleton/actin organization and regulation of GTPase activity as clearly enriched terms but also identified vesicle-mediated transport and endocytosis as highly significant hits (Fig. 1; Table S7). The AA-regulated phosphoproteins associated with these GO terms are listed in Table S8.

**Figure 2:**
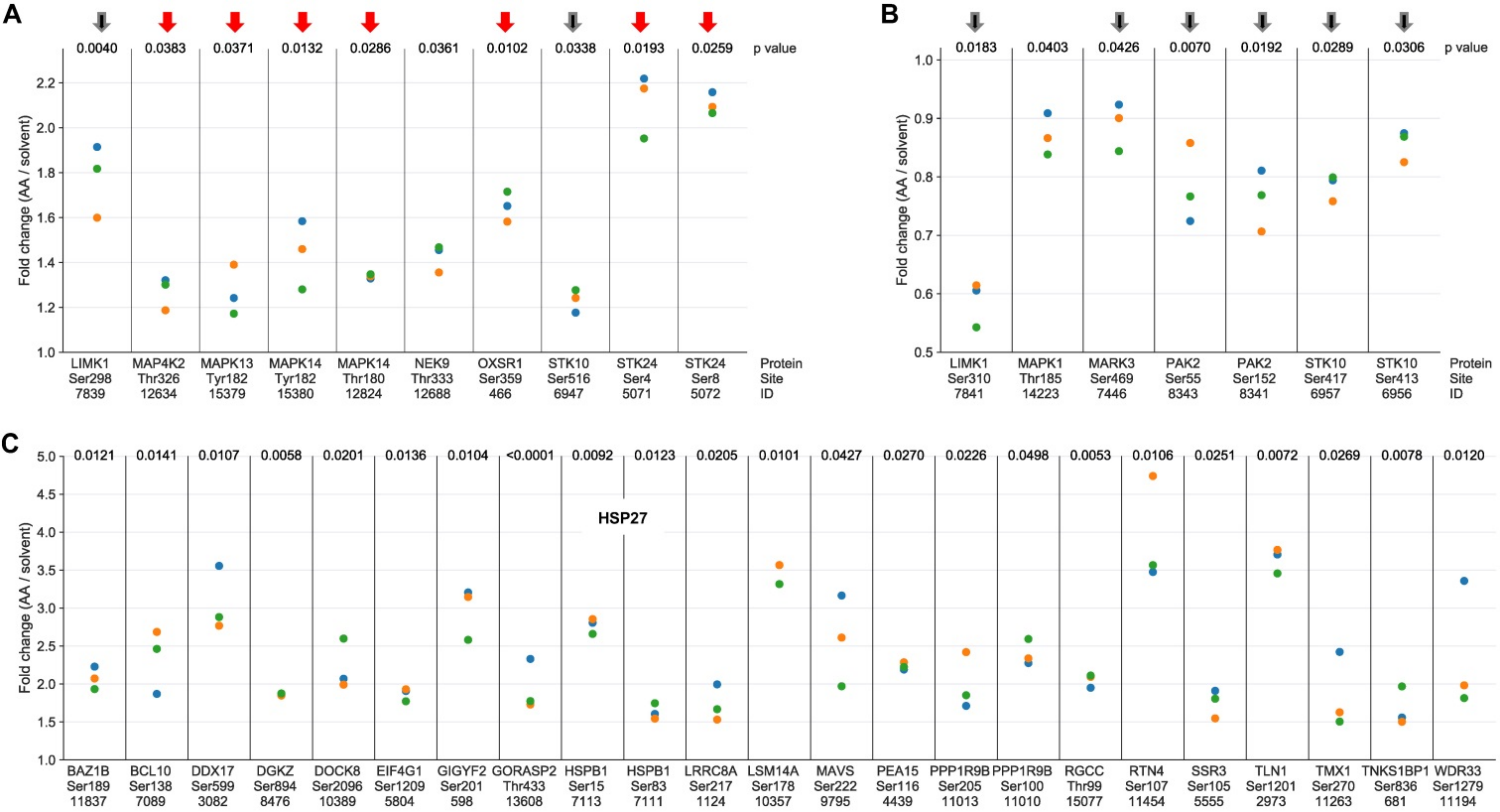
** Phosphosites showing a significant response to AA treatment in MDMs.** (A) Protein kinases with phosphosites upregulated by AA versus solvent (*p <* 0.05 by paired t test; FC > 1.1; n = 3 biological replicates) identified by phosphoproteome analysis. (B) Protein kinases with phosphosites downregulated by AA (*p <* 0.05; FC < 0.9; n = 3). Red arrows: protein kinases linked to stress response; black arrows: protein kinases associated with actin filament organization. (C) Phosphosites in proteins other than protein kinases strongly upregulated in response to AA treatment ( *p <* 0.05; FC > 1.7; n = 3). ID refers to the peptide identified by MS in Table S3. Fold change values were calculated by dividing normalized signals (see Materials and Methods). Data points represent individual samples.

The AA-mediated phosphorylation of protein kinases linked to stress response (red arrows) or actin filament organization (black arrows) is illustrated in Figure 2A (upregulated phosphosites) and Fig. 2B (downregulated phosphosites). In line with current knowledge, the stress response proteins include two members of the p38 family, p38δ (HUGO name: MAPK13) and p38α (HUGO name: MAPK14) as well as their upstream regulator mitogen-activated protein kinase kinase kinase kinase 2 (MAP4K2) and serine/threonine kinase 24 (STK24) (Fig. 2A), suggesting a central role for p38 in AA-mediated signaling. Protein kinases of the second group (black arrows) mainly encompass components of Rho family GTPase-regulated signal transduction pathways regulating actin filament organization 53, i.e., LIM domain kinase 1 (LIMK1), serine/threonine kinase 10 (STK10) and p21Rac1 activated kinase 2 (PAK2), consistent with a role for AA-signaling in actin reorganization. Both, LIMK1 and STK10 contain phosphosites that are affected by AA in opposite ways, which may indicate opposite regulatory effects of these sites (inhibitory or stimulatory).

The notion that AA-signaling impacts actin reorganization is supported by AA-mediated effects on a large number of other phosphoproteins with known functions in actin filament organization, as exemplified for AA-upregulated phosphosites in Fig. 2C. Intriguingly, these include two sites in heat shock protein 27 (HSP27; HUGO name: HSPB1), which is a direct substrate of p38 and a regulator of actin filaments 54, thus suggesting a potential link of AA-regulated signal transduction and biological functions. This question is addressed further below.

### Transcriptional profiling of AA-treated MDMs

**Figure 3:**
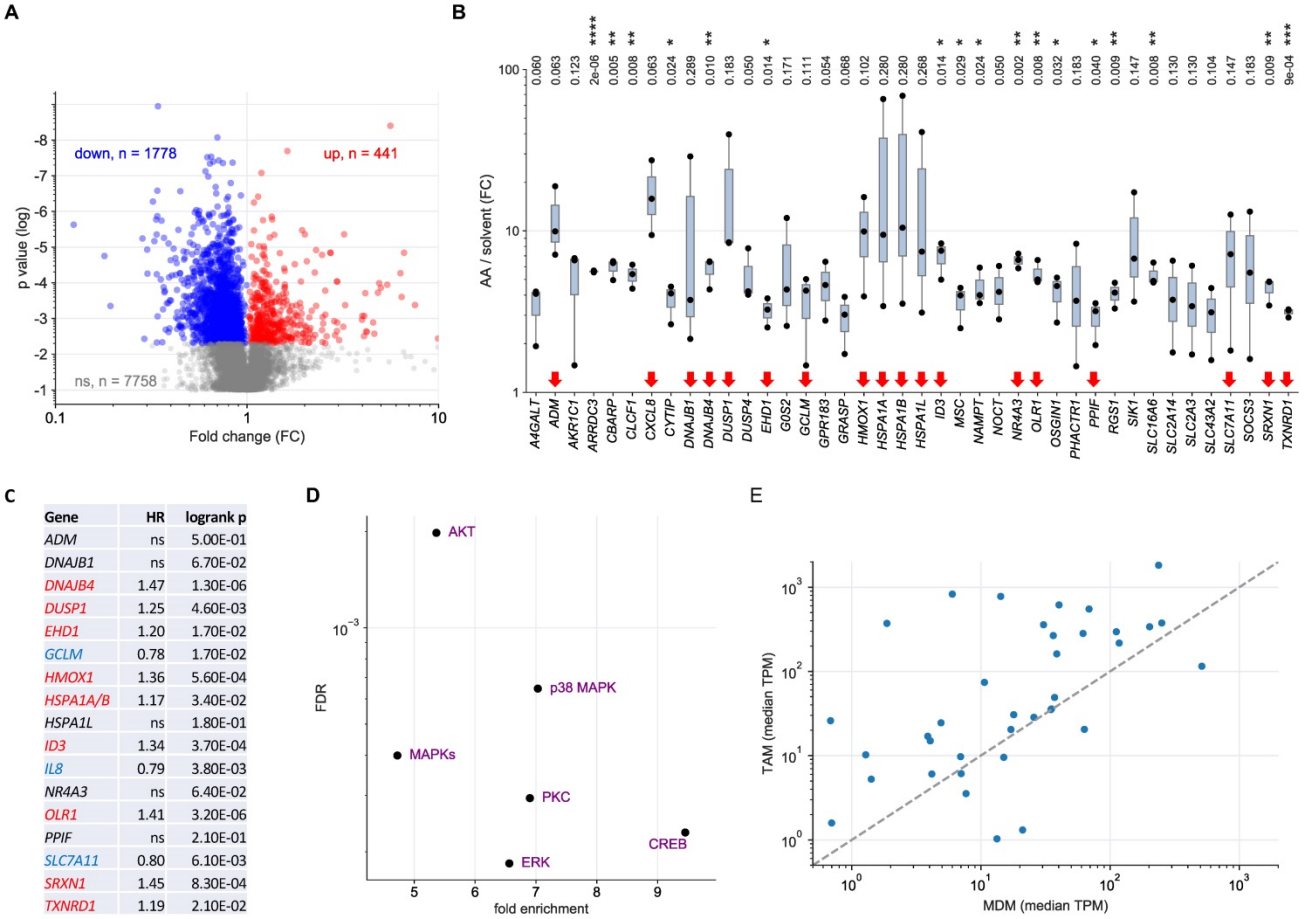
** Transcriptional profiling of AA-treated MDMs and *ex vivo* TAMs.** MDMs were treated with 50 µM AA or solvent for 3 h and RNA was analyzed by next-generation sequencing (RNA-Seq) for 3 independent donors. (A) Volcano plot depicting genes significantly (nominal *p <* 0.05) repressed by AA (blue), induced (red) by AA) or showing no significant change (grey). (B) RNA-Seq results for the top AA-induced genes (FC > 3; TPM > 3 in AA-treated cells). Red arrows: genes linked to stress response according to GO term enrichment analysis (GO:0006950 in Table S6; genes in Table S8). Box pots show medians (line), upper and lower quartiles (box) and range (whiskers). Significance was tested by paired t test: **** *p <* 0.0001; *** *p <* 0.001; ** *p <* 0.01; * *p <* 0.05 for AA versus solvent; p values were adjusted for multiple hypothesis testing by Benjamini Hochberg correction. (C) Association of the expression of stress-response-linked genes (red arrows in panel B) with relapse-free survival of serous ovarian cancer according to the Kaplan-Meier Plotter database (see Materials and Methods for details; *HSPA1A* and *HSPA1B* are combined in panel C; *CXCL8* is referred to as *IL8* in panel C). Red letters: positive hazard ratio (HR); blue: negative HR; ns: not significant logrank p value (≥0.05). (D) Ingenuity Upstream Regulator Analysis of the AA-induced genes identified in panel A. The plot shows the regulators with the lowest false discovery rate (FDR: < 0.05) and the strongest enrichment (> 3-fold). (E) Comparison of upregulated genes in MDMs and TAMs (TPM > 1 in TAMs) in an independent dataset (RNA-Seq; see Table S11 for details). The Pearson correlation coefficient for the data points shown is r = 0.32 (p = 0.052).

To determine the AA-regulated transcriptome of MDMs we performed RNA-Seq analyses of 3 biological replicates of cells treated with 50 µM AA or solvent for 3 h (Table S9). As shown by the volcano plot in Fig. 3A, 441 genes were significantly upregulated and 1,778 genes significantly downregulated by AA compared to solvent (nominal *p* value < 0.05 by *t* test). The most robust group of AA-induced genes (n = 40; Fig. 3B), i.e., showing a minimum fold change (FC) of 3 and a minimum expression of 3 transcripts per million (TPM), were further analyzed by functional annotations. GO term enrichment analysis identified response to oxidative stress as the most significant and highly enriched specific category, followed by apoptosis-related terms (Table S10). Genes associated with response to stress comprised 45% (n = 18) of all genes analyzed (red arrows in Fig. 3B), supporting the relevance of these genes with respect to AA-induced signaling. Intriguingly, the expression of 9 of these 18 genes in serous OC is associated with a poor RFS (Fig. 3C) according to data in the Kaplan-Meier Plotter databases (https://kmplot.com) 51, pointing to a possible connection of the AA-induced stress response to tumor progression.

Ingenuity Pathway Analysis (IPA) of AA-induced genes identified MAP kinase signaling (including p38) as the most significant upstream regulators (Fig. 3D). Other enriched upstream regulators were cAMP Responsive Element Binding Protein (CREB), a known p38 target 55, as well as protein kinase C (PKC) and AKT pathways, which are also linked to MAPK signaling by various interconnections 56, 57. Taken together, these results in conjunction with the results of the phosphorproteome analysis clearly identify a p38-mediated stress response, including a specific transcriptional program, as a major component of AA-induced signaling. On the other hand, transcriptional profiling did not reveal any evidence for actin-mediated effects, suggesting that Rho-family-GTPase-related signaling events via actin filaments are relevant primarily with regard to non-transcriptional mechanisms. We also identified 23 “robustly” downregulated genes (FC< 0.33; TPM > 3; Fig. S1), but functional annotation did not yield any significant conclusion.

To assess the relevance of these findings in the context of the OC microenvironment we compared the expression of the upregulated genes in MDMs and TAMs from OC ascites (Table S11; TPM > 1 in TAMs). As illustrated in Fig. 3E, the vast majority of these genes (30/37; 81.1%) were expressed at higher levels in TAMs compared to MDMs (median TPM in TAMs > 1), suggesting that AA affects a similar set of genes in TAMs *in vivo* and MDMs *in vitro*.

### Induction of ASK1-p38 signaling by non-metabolized AA

**Figure 4:**
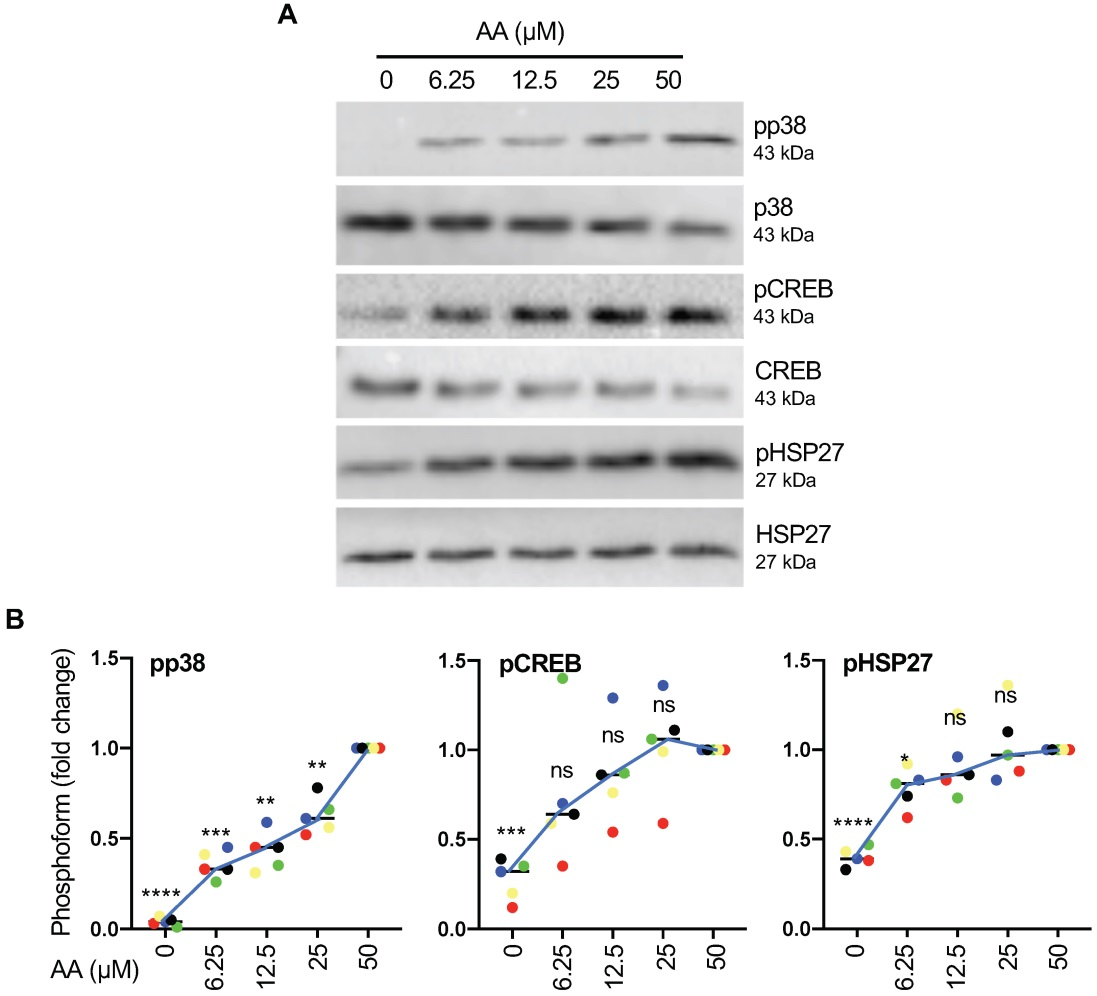
** Signaling events induced by AA in MDMs.** (A) Dose-dependent induction of p38, HSP27 and CREB phosphorylation by AA analyzed by immunoblot analysis. MDMs were incubated with different concentrations of AA for 30 min. Depicted are representative immunoblots. (B) Quantification of n = 5 biological replicates. Data points represent individual samples.The lines connect the median values depicted as horizontal lines. Statistical analysis was performed by paired t test and p values were adjusted by Benjamini-Hochberg correction. **** adjusted *p <* 0.0001; *** *p <* 0.001; ** *p <* 0.01; * *p <* 0.05 against samples treated with 50 µM AA. ns: not significant. Values represent the fold change relative to 50 µM.

To understand the regulation by AA in more detail we next analyzed the connection of p38 phosphorylation to other components of the p38 signaling cascade. To this end, we first analyzed the dose dependence of p38 phosphorylation (Thr-180, Tyr-182) on the p38 substrates HSP27 (Ser-82) and CREB (Ser-133) by immunoblotting. Figs. 4A and B show that p38 phosphorylation increased nearly linearly within the tested concentration range of 6.25 - 50 µM of AA, while CREB and HSP27 phosphorylation reached saturation below 50 µM. This suggests that maximum levels of activated p38 (pp38) are not required for full phosphorylation of CREB and even less so for HSP27 phosphorylation. However, in view of maximum phosphorylation p38 being observed at 50 µM AA, which is close to the median level found in OC ascites 19, we chose this concentration for all subsequent experiments.

**Figure 5:**
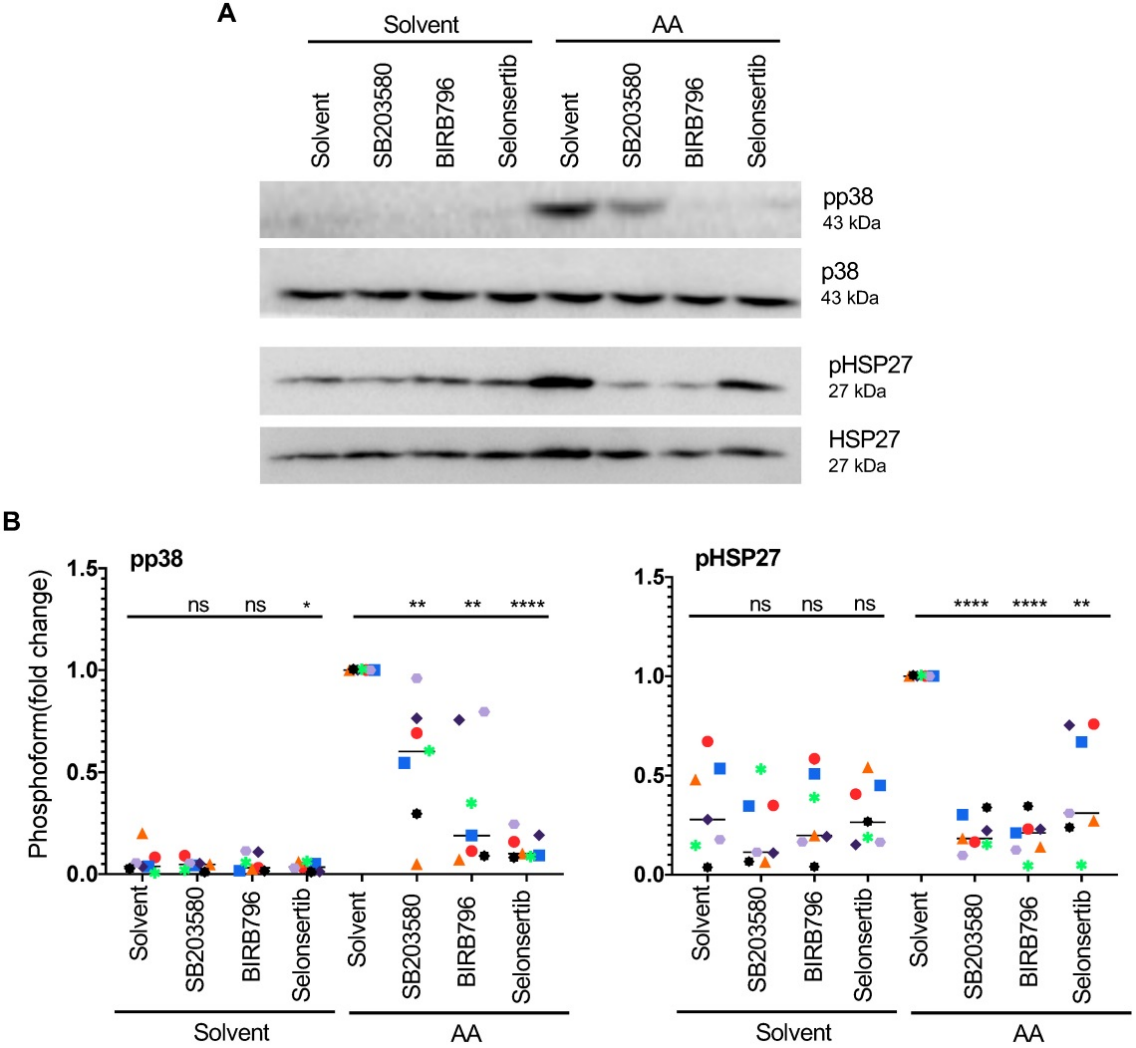
** Effect of pharmacological inhibition of ASK1 or p38 on AA-induced HSP27 phosphorylation.** (A) Immunoblot of MDMs pretreated with the selective ASK1 inhibitor Selonsertib (1 µM) or the p38 inhibitors BIRB796 or SB203580 (10 µM) for 1 h prior to treatment with AA for 30 min. (B) Quantification of n = 7 biological replicates. Data points represent individual samples, horizontal lines indicate the median. Statistical analysis was performed by paired t test. **** adjusted *p <* 0.0001; *** *p <* 0.001; ** *p <* 0.01; * *p <* 0.05 against samples treated with AA + solvent. Detection of pp38 was not reliably possible in solvent only samples. ns: not significant. Values represent the fold change relative to AA + solvent.

The phosphorylation of HSP27 in response to AA is of particular interest in view of a reported role for HSP27 in actin filament reorganization. To analyze the AA-induced pathway leading to HSP27 activation in more detail, we investigated the effect of pharmacological inhibitors. As shown in Fig. 5A and B, both p38 inhibitors tested, SB203580 and BIRB796 strongly diminished AA-induced p38 autophosphorylation and HSP27 phosphorylation. A well-known activating kinase upstream of p38 is apoptosis signal regulating kinase 1 (ASK1; HUGO name: MAP3K5). Selonsertib, a small-molecule clinically tested inhibitor of ASK1 58, 59, also inhibited AA-induced p38 and HSP27 phosphorylation by 92% and 69%, respectively, thus confirming the AA-mediated induction of an ASK1 → p38 → HSP27 signaling pathway.

**Figure 6:**
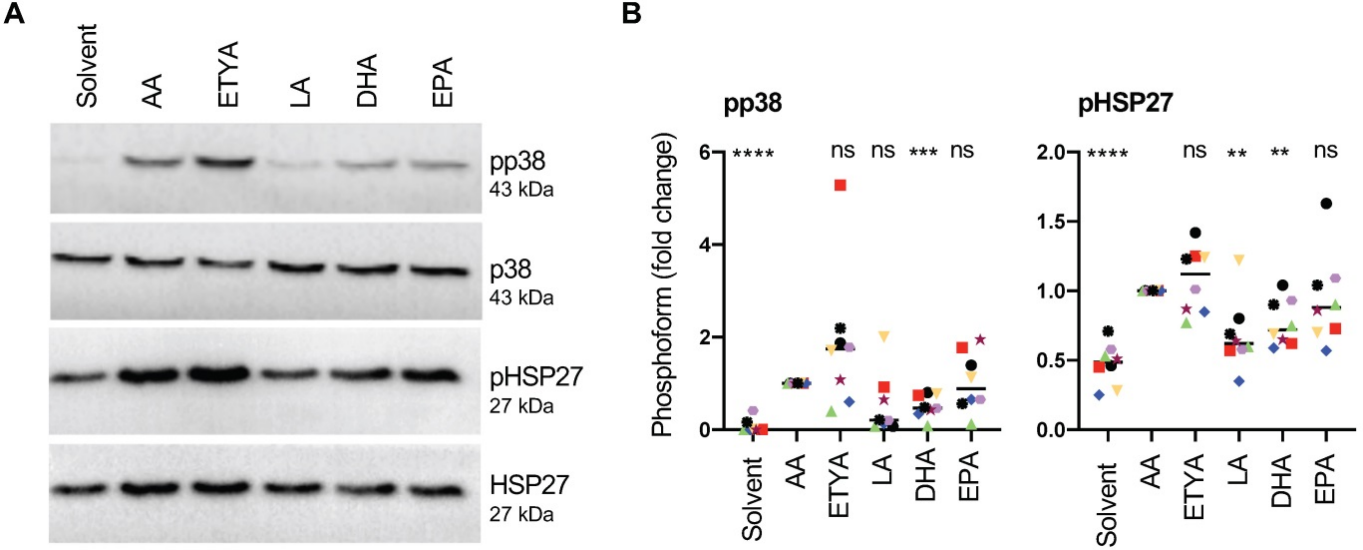
** Signaling events induced by AA in MDMs compared to other PUFAs.** (A) Immunoblot analysis of p38, HSP27 and CREB phosphorylation in response to 50 µM AA compared to 50 µM ETYA, LA, DHA or EPA (representative example). Details as in Fig. 4. (B) Quantification of n = 8 biological replicates. Data points represent individual samples, horizontal lines indicate the median. Statistical analysis was performed as in Fig. 4. Values represent the fold change relative to AA.

Next, we asked whether these signaling events are triggered by non-metabolized AA or an eicosanoid generated by the cyclooxygenase (COX) or lipoxygenase (LOX) pathways. Figs. 6A and B unequivocally show that the non-metabolizable AA-analogue and dual COX/LOX inhibitor 5,8,11,14-eicosatetraynoic acid (ETYA) 60 induced p38 and HSP27 phosphorylation to a very similar extent as AA, indicating that metabolizing AA is not required.

We also tested the effect of other structurally related PUFAs and found that linoleic acid (LA), eicosapentaenoic acid (EPA) and docosahexaenoic acid (DHA) were also able to induce p38 phosphorylation. While the median effect of EPA approached that of AA and ETYA (89% versus AA), LA and DHA induced p38 phosphorylation to a considerably weaker extent (LA: 20.5%; DHA: 47%; Figs. 6A and B). HSP27 phosphorylation was also induced by all PUFAs, closely following the pattern observed for pp38 (Figs. 6A and B). The observed phosphorylation of p38 by PUFAs other than AA is consistent with the conclusion that this AA-mediated effect is triggered by AA itself rather than its metabolites.

Collectively, these observations indicate that induction of p38 → HSP27 signaling is selective, but not specific for AA, and is not dependent on the metabolization of AA.

### Role of Ca^2+^-mediated signal transduction pathways in the AA-induced activation of p38

**Figure 7:**
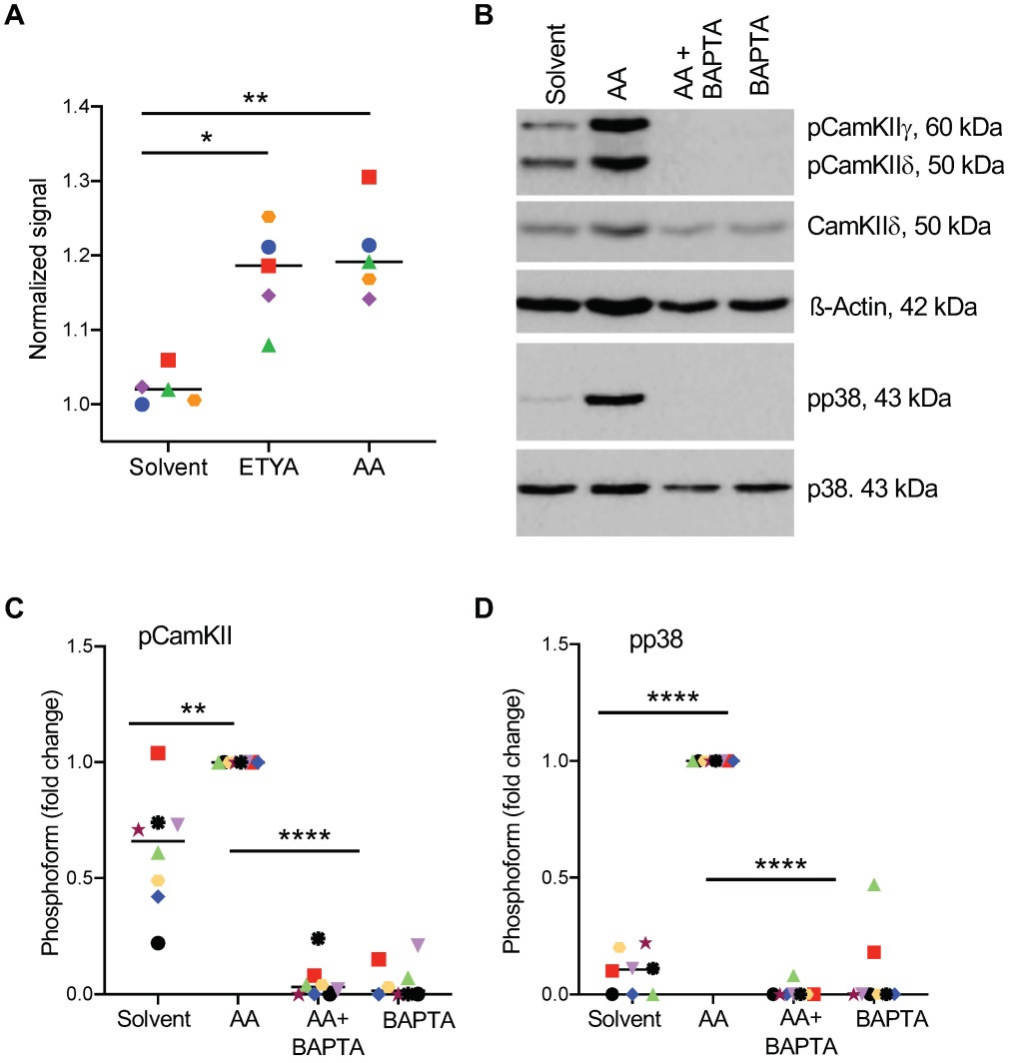
** Effect of Ca^2+^-dependent signaling on AA-mediated phosphorylation of p38.** (A) Increase in intracellular Ca^2+^ levels 0-105 sec after stimulation with 50 µM AA or ETYA. Kinetic measurements of Fura-2 fluorescence were carried out as described in Materials and Methods. (B) AA-mediated phosphorylation of p38 and CAMK IIγ and δ, and its inhibition by the Ca^2+^ chelator BAPTA-AM. The cells were pretreated with 50 µM BAPTA-AM for 1 h prior to treatment with 50 µM AA for 30 min. (C, D). Quantification of 8 biological replicates as in panels A and B. Data points represents individual samples, horizontal lines indicate the median. Statistical analysis was performed by paired t test: **** adjusted *p <* 0.0001; *** *p <* 0.001; ** *p <* 0.01; * *p <* 0.05 against AA-treated samples.

To elucidate the AA-triggered signaling event upstream of p38, we analyzed the role of Ca^2+^-mediated signaling previously implicated in conveying AA-triggered signals in rat neutrophils 29. As shown in Figs. 7A and S2, both AA and ETYA stimulation of MDMs induced a rapid cytoplasmic influx of Ca^2+^ and phosphorylation of calcium/calmodulin-dependent protein kinase II (CAMKII; HUGO name: CAMK2; Fig. 7B), known as a direct activator of ASK1 61, 62. Consistent with these findings, sequestration of Ca^2+^ by the calcium chelator BAPTA-AM (1,2-bis(o-aminophenoxy)ethane-N,N,N′,N′-tetraacetic acid) 63 blocked the AA-triggered phosphorylation of p38 (Fig. 7B). A quantification of these data is shown in Fig. 7C and D.

To elucidate the potential origin of Ca^2+^ impinging on the CAMKII - ASK - p38 axis, we tested the effects of the FFAR4 antagonist AH7614, the PLCγ inhibitor U-73122, the G_i/0_ inhibitor pertussis toxin and the G_q/11_ inhibitor YM-254890. These compounds partially inhibited the AA-induced phosphorylation of p38 in MDMs from some donors, while no effect was detectable in others (Fig. S3). These findings suggest that an AA → GPCR/FFAR4 →PLCγ pathways triggers the release of Ca^2+^ from intracellular stores via inositol trisphosphate in a subset of donors to a minor extent, suggesting that a GPCR-/PLCγ-independent mechanism(s) plays a more prominent role in transducing AA-elicited signal to p38.

**Figure 8:**
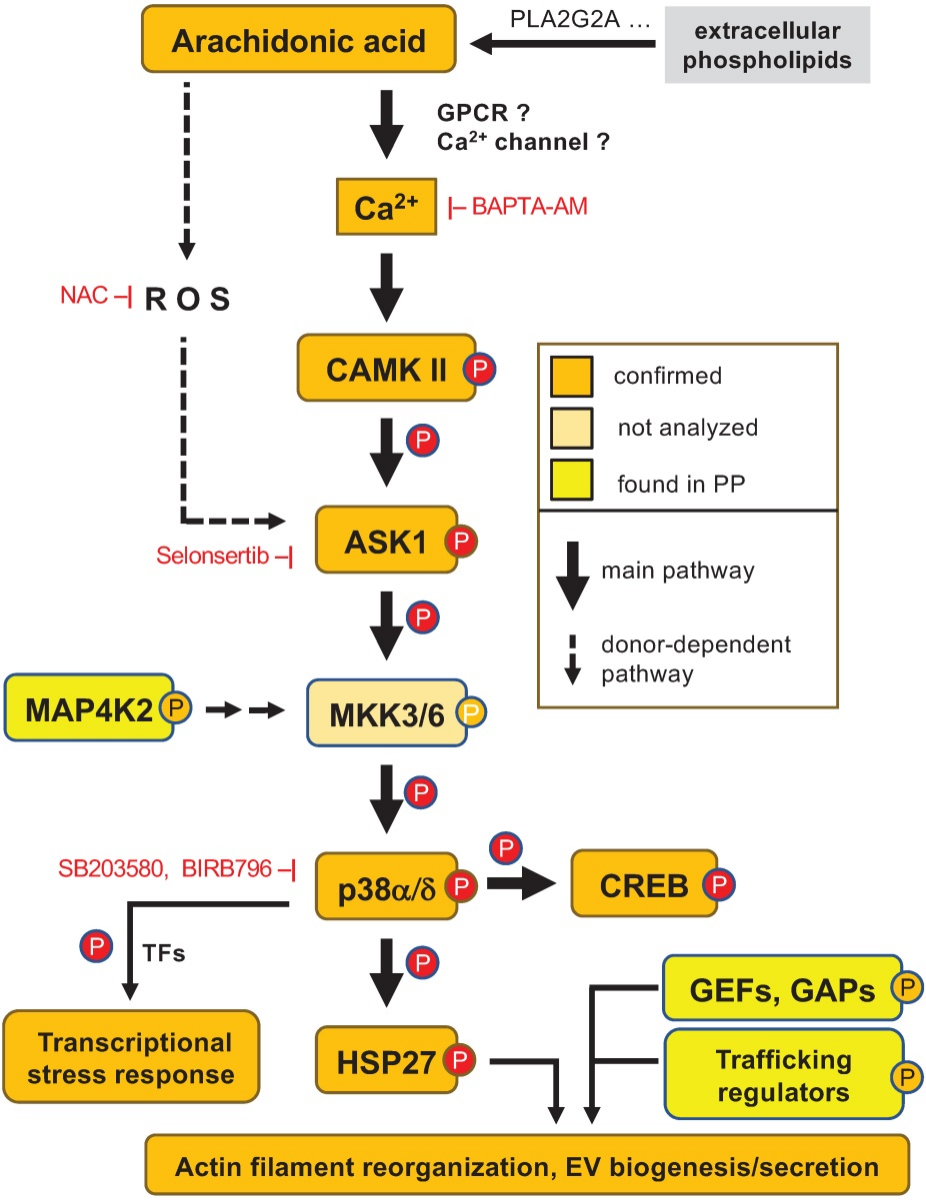
** Model of AA-regulated signal transduction pathways.** An AA-triggered Ca^2+^- ASK1 - p38 pathway induces a transcriptional stress response and mediates HSP27 phosphorylation, thereby contributing to actin filament reorganization in concert with AA-regulated RhoGEFs and RhoGAPs. Other protein kinases phosphorylated in response to AA, such MAP4K2, may also impinge on this pathway. ROS produced by different mechanisms may contribute to p38 phosphorylation to a minor extent in a subset of donors. Orange: phosphosites identified by MS and confirmed by immunoblotting; yellow: phosphosites identified by MS. EX: extracellular space; PM: plasma membrane; CYT: cytosol; NUC: nucleus. AA is most likely derived from extracellular phospholipids with PLA2G2A possibly playing a crucial role (see text and Table 1).

Based on this data AA-triggered signal transduction is initiated by multiple, donor-dependent events (including GPCR(s) and PLCγ), which merge on a common Ca^2+^-regulated pathway: Ca^2+^ → CAMK II → ASK1 → (MKK3/6) → p38 → HSP27/CREB (parentheses: not analyzed in the present study; see scheme in Fig. 8).

### Role of ROS in AA-triggered p38 phosphorylation

Next, we addressed the potential role of ROS in light of previously published findings suggesting the generation of intracellular ROS in response to AA 20, 21. Fig. S4 shows an example of AA-mediated induction of total ROS and superoxide generation in one MDM sample. However, AA-induced ROS production varied strongly among individual MDM samples and, despite a similar AA-induced p38 phosphorylation across all samples was even undetectable in most cases (Fig. S5). Furthermore, quenching of ROS by N-acetylcysteine (NAC) or the NOX inhibitor VAS3947 only partially affected p38 phosphorylation, if at all (Fig. S5). We conclude from these observations that ROS production, if any, plays a secondary role as a mediator of AA-induced p38 activation in a subset of donors (Fig. 8).

### Interference of AA with actin filament-dependent processes

**Figure 9:**
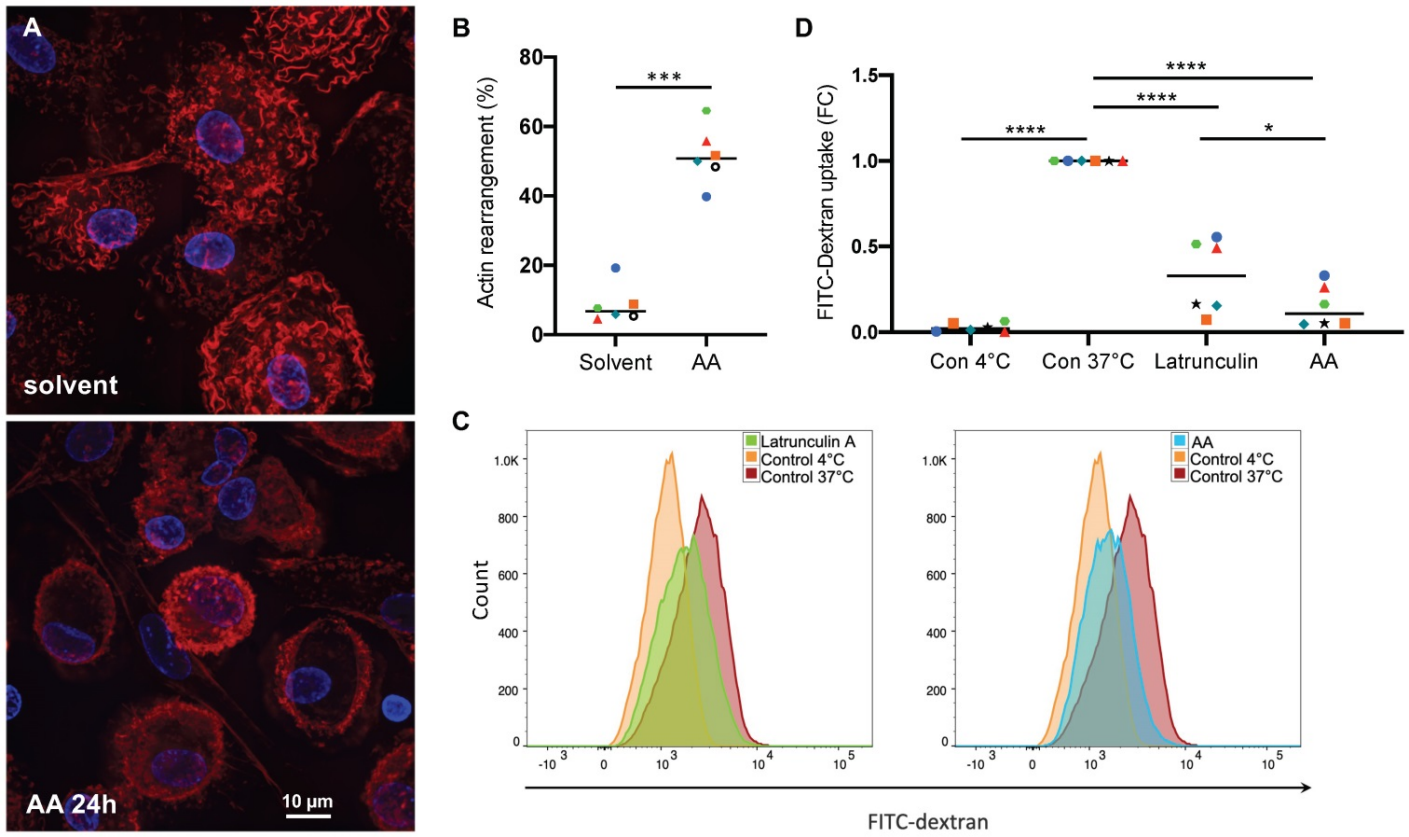
** Effect of AA on actin filament organization and macropinocytosis.** (A) Phalloidin staining of actin filaments in MDMs treated with 50 µM AA or solvent (control) for 24 hrs (representative experiment). (B) Quantification of actin filament rearrangement in MDMs from n = 6 donors after treatment as in panel A (each donor is represented by a specific symbol). Cells were counted as positive for actin rearrangement, if the typically wavy actin fibers were undetectable and/or actin filaments were accumulated at the cell edges. For each sample, on average 70 cells were evaluated. (C, D) Flow cytometric analysis of FITC-Dextran pinocytosis by MDMs treated with 50 µM AA for 24 h. Latrunculin A was included as a known pinocytosis inhibitor 67. Untreated MDMs incubated on ice to invoke a complete inhibition of pinocytosis were used as negative control. Significance was tested by paired t test: ****p < 0.0001, ***p < 0.001, * *p <* 0.05. The gating strategy is shown in Fig. S6.

As shown above, AA affects the phosphorylation status of the pp38 substrate HSP27 as well as multiple regulators of the Rho family GTPases (Figs. 1 and 2), pointing to an impact on actin filament organization. To test this hypothesis we stained MDMs with fluorescently labeled phalloidin (California red conjugate) as an actin-binding probe 64. Figs. 9A shows actin fibers evenly distributed in solvent-treated cells, which was altered in 40-65% of cells (Fig. 9B) after a 24-hr incubation with 50 µM AA, with actin staining largely restricted to the cell edges and/or reduced to a low intensity, suggesting that AA prevents the assembly or promotes the disassembly of actin filaments. In view of these findings, we studied the effect of AA on macropinocytosis as an actin-driven process 65. As shown in Figs. 9C and D AA inhibited the uptake of FITC-labelled dextran by MDMs by 91% after 24 hrs incubation. This inhibitory effect was even stronger than that of Latrunculin A, which sequesters monomeric G-actin and prevents actin filament assembly 66, thereby blocking endocytotic uptake by pinocytosis or phagocytosis 67, 68. Taken together, these findings confirm that AA-induced signaling interferes with endocytotic uptake functions of MDMs by interfering with actin filament organization.

### Impact of AA on the release of EVs

**Figure 10:**
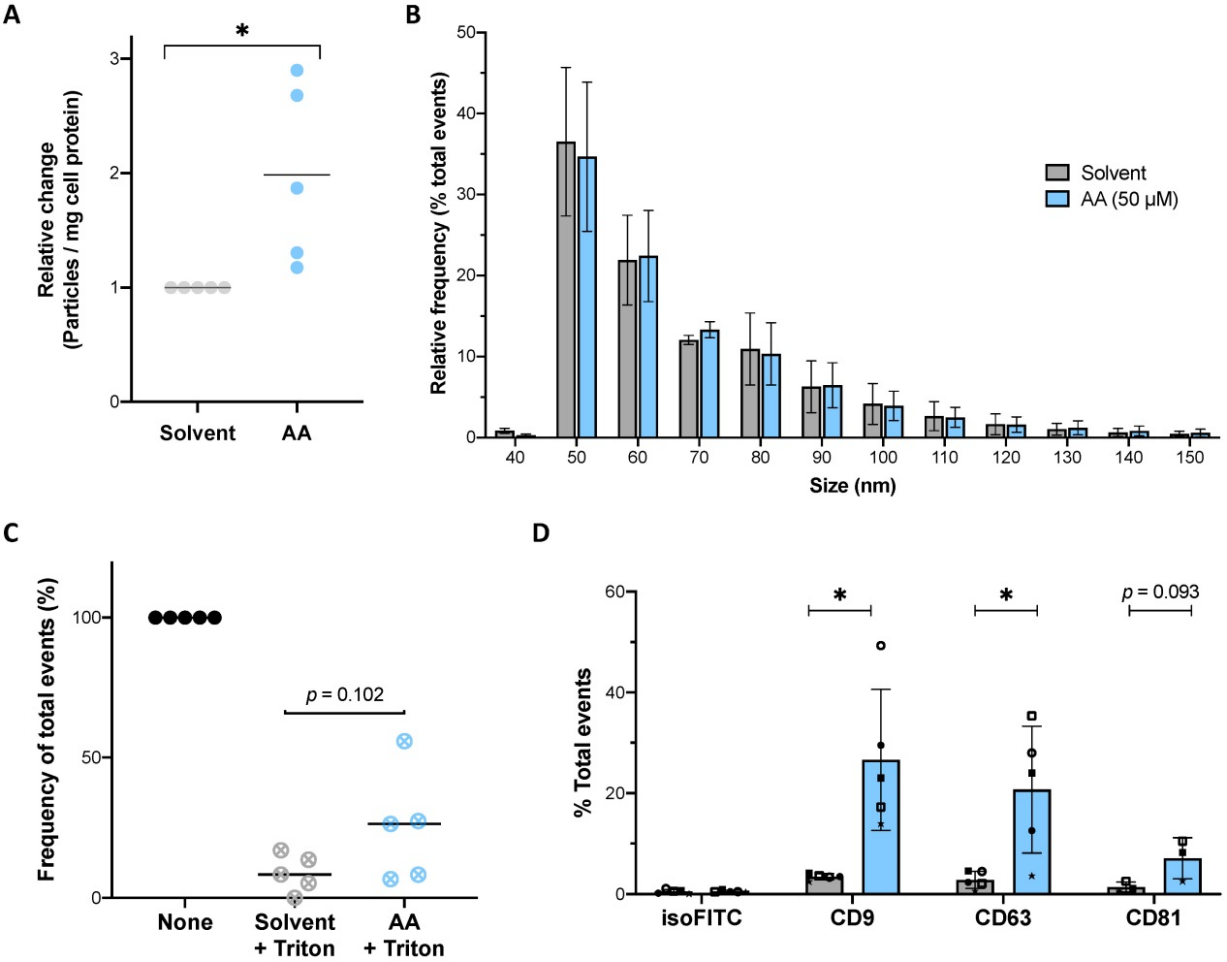
** Effect of AA on the release of EVs.** (A) Relative number of particles released by MDMs into the culture medium after 24 h of treatment with AA (blue dots) or solvent (grey dots) determined by HSFC. Values were calculated per mg total cell protein and normalized to 1 for solvent-treated cells. (B) Size distribution of particles released by MDMs shown in panel A. Histogram of particle size with a bin width of 10 nm for AA (blue bars) and solvent control (grey bars) samples. Data were normalized to the total number of acquired events separately for solvent and AA-treated samples. The plot shows the mean (± SEM) of the relative frequency of total events detected by HSFC in conditioned media from n = 5 donors. (C) Relative decrease of particle count in conditioned media after treatment with Triton X-100 (final concentration 0.05%). Values obtained before addition of detergent were set to 100%. (D) Immunostaining results on EV pellets obtained by differential centrifugation. The presence of tetraspanin markers (CD9, CD63 and CD81) was evaluated on particles obtained from n = 3-5 donors. A histogram of particle size of positive events is shown in Figure S8 for a representative sample. Bars represent mean (± SD) of total % of positive events. Symbols represent the values obtained with independent donors. Significance was tested by paired t test: * *p <* 0.05 for AA versus solvent.

The functional annotation analysis (Fig. 1 and Table S7) identified a strong association of AA-downregulated phosphosites with vesicle-mediated transport. We therefore analyzed the effect of AA on the release of extracellular vesicles (EVs) by MDMs. High-sensitivity flow cytometry (HSFC) analysis of conditioned medium from MDMs cultured in the presence of 50 µM AA under serum-free conditions revealed a significant approximately 2-fold increase in the frequency of released particles (Fig. 10A) within a size range characteristic of EVs 49, 69 (~90% ranging from 50 to 100 nm; Fig. 10B). These particles were highly sensitive to detergent treatment, consistent with their classification as EVs (Fig. 10C). We also quantified EVs isolated by differential ultracentrifugation from conditioned media, which confirmed a higher release from AA-treated MDMs (average 2.94 × 10^9^ particles, n = 9 donors) compared to solvent controls (average 1.61 × 10^9^ particles) (relative change illustrated in Fig. S7). To further characterize these samples, we applied flow cytometry to analyze the expression of the tetraspanins CD9, CD63 and CD81 as classical exosomal markers 70, 71. This analysis showed positive events for all markers in both AA-treated and solvent controls with a 5- to 8-fold increase in samples from AA-induced MDMs (Figs. 10D and S8). In conjunction with the results of the size analyses (Fig. 10B), these findings confirm the released particles as *bona fide* EVs with exosomal features. These observations also lend strong support to the hypothesis that AA-mediated signaling events affect proteins that regulate the biogenesis and/or release of tetraspanin-containing EVs as suggested by our phosphoproteomics results.

### Expression and survival association of AA-generating phospholipase A2 isoforms

The association of both AA 13 and AA-induced genes (Fig. 3C) with a poor clinical outcome suggests that decreased AA levels could be a therapeutically beneficial achievement. AA is generated by the phospholipase A_2_-mediated cleavage of both extra- and intracellular phospholipids 72-74. Phospholipase A_2_ enzymes are encoded by 21 different *PLA2* genes, whose contribution to OC progression is poorly understood. To identify potential therapeutic targets we analyzed the expression of *PLA2* genes in solid tumor samples from OC patients (which contain both tumor and tumor-associated host cells as possible cellular sources of PLA2) and their association with the clinical outcome. Intriguingly, analysis of the data in the PRECOG database revealed that within this gene family, only *PLA2G2A* was associated with the overall survival (OS) of OC with near-significance (Table 1; z-score = 1.93). Consistently, *PL2G2A* was also associated with a shorter progression-free survival of high-grade serous OC bases of the data in the Kaplan Meier Plotter meta-analysis database (Fig. S9). In agreement with these findings, *PLA2G2A* showed the highest variance of expression in solid OC tumor samples (Table 1). Furthermore, *PLA2G2A* exhibited the highest maximum expression among all *PLA2* family members (Table 1). These findings suggest that the PLA2G2A (PLA_2_ Group 2A) isoform plays a crucial role in the generation of clinically relevant AA, and thus represents an interesting target for pharmacological intervention with OC progression.

## Discussion

The present study is based on our original discovery that the concentration of AA in OC ascites is associated with short RFS 13, for which the underlying molecular mechanisms remeined unknown. TAMs represent the major host cell population in ascites, and in view of their link to a poor prognosis are of particular interest in this context. In the present study, we therefore asked the question, which signal transduction pathways and biological functions are regulated by AA in macrophages. We addressed this problem by a combination of omics, biochemical and functional analyses, which revealed a comprehensive picture of AA-regulated signal transduction pathways in MDMs and identified AA-affected biological functions potentially relevant in the TME.

### The AA-triggered p38 signaling pathway

Our phosphoproteomics, transcriptomics and complementary immunoblotting analyses revealed the stress-response-associated p38 MAP kinase pathway as a central target of AA signaling in MDMs (Figs. 1-4). Consistent with this finding we identified the stress-response kinase ASK1 75-77 as the upstream regulator of p38 (Fig. 5), presumably via MKK3/6 78. In agreement with this finding, the known p38 substrates CREB and HSP27 55 were also phosphorylated in response to AA in an ASK1-dependent manner (Fig. 5). Importantly, these results were confirmed with the non-metabolizable AA analog and COX/LOX inhibitor ETYA 60 (Fig. 6), indicating that the observed effects were caused by AA itself rather than its metabolites. This is consistent with the observation that other PUFAs triggered the effects as well, albeit to a reduced extent (Fig. 6).

Even though redox signaling has been reported as the main activator of ASK1 in other experimental systems 79-81, this was not the case in AA-stimulated MDMs. AA-induced ROS production was observed in MDMs only from a subset of donors, and even in positive cases its contribution to p38 phosphorylation was low (Figs. S4 and S5). We therefore analyzed CAMK II as another known direct activator of ASK1 61, 62. Our data clearly show that CAMK II phosphorylation was induced by AA in all MDM samples analyzed. Consistent with this observation, AA induced a rapid accumulation of intracellular Ca^2+^, and the Ca^2+^ chelator BAPTA-AM 63 prevented CAMK II and p38 phosphorylation (Figs. 7A-C).

Others have reported that GPCR-mediated signaling and PLC are instrumental in AA-triggered signaling in rat neutrophils, but we were unable to confirm this for MDMs. The pharmacological inhibition of PLC, FFAR4 or GPCR-associated G proteins reduced p38 phosphorylation only in a subset of samples, and if so, only to a low extent (< 50%; Fig. S3). This suggests that GPCR-mediated PLC activation only plays a minor role in the intracellular accumulation of Ca^2+^ and the ensuing activation of CAMK II in AA-stimulated MDMs. It is possible that lipid membrane structures associated with Ca^2+^ channels are affected by AA in MDMs, as reported for other experimental systems 82-86, potentially resulting in increased intracellular Ca^2+^ levels in AA-treated cells. Taken together, these findings indicate the following pathway to play a major and donor-independent role in AA-stimulated MDMs: AA → multiple mediators → Ca^2+^ → CAMK II → ASK1 → MKK3/6 → p38 → HSP27 / CREB.

### AA signaling impinges on actin-dependent processes

GO term enrichment analyses of our phosphoproteomics data identified several potentially interesting functions affected by AA apart from “response to stress”, one of which is “actin cytoskeletal organization” (Fig. 1; Tables S4 and S5). In agreement with this finding, an enrichment was also found for the term “GTPase signaling”, representing a group of G protein regulators [guanine nucleotide exchange factors (GEFs) and GTPase activating proteins (GAPs), Table S8] impacting Rho/Rac pathways and thereby actin cytoskeletal structures 53. At present, little is known about the precise function of most of these proteins, in particular with respect to the functional consequences of their phosphorylation, complicating the interpretation of these findings. This applies in particular to the observation that several proteins, including LIMK1 and STK10, contain phosphosites that are upregulated by AA, while other sites in the same protein are downregulated. It may be speculated that such modifications exert opposite regulatory effects, but this question may only be clarified once the functions of these proteins and their regulation by phosphorylation are known.

Another interesting aspect in view of the observed AA-induced morphological changes is a potential involvement of signaling pathways upstream of myosin II. While the myosin light chain kinase MLCK was not detected in the MDM proteome (Table S3), two other proteins phosphorylated in response to AA may be of interest in this context, i.e., MYH9 and MYO9B (Table S4). MYO9B is an unconventional myosin involved in intracellular movements. It interacts with actin, is inhibited by Ca^2+^, figures as a RhoA GTPase activator and plays a role in cell migration 87, 88. Furthermore, the AA-induced phosphorylation of MYO9B at S1354 found in the present study was also observed in platelets, where MYO9B phosphorylation mediated by PKC and PKD increased its RhoA GTPase activity 89.

MYH9 is a non-muscle myosin IIA heavy chain, which is involved in the generation of intracellular mechanical force and translocation of the actin cytoskeleton 90. MYH9 is phosphorylated by AA in MDMs at S1943 (Table S4), a site described to regulate cellular protrusions and invasion 91. Taken together, these observations raise the possibility that the observed AA-induced phosphorylation of MYH9 and MYO9B play a role in mediating the observed morphological changes.

In agreement with these findings and the predictions of the GO term enrichment analysis, we found microscopic evidence for an impact of AA on actin organization, indicating an interference with the assembly of actin filaments and the formation of an actin ring at the cellular edges (Figs. 9A and B). This observation is consistent with previous reports describing AA-induced actin ring formation in osteoclasts 92, and actin clustering at the cell membrane of MDMs to be regulated by external signals impacting their polarization 93. AA had no cytotoxic effects on CD14+ monocytes after 48 hrs of exposure (tested for up to 80 µM) 92, and cytotoxicity affected only few cells in our own experiments (as indicated by normal nuclear morphology and a low fraction of detached cells after AA treatment), ruling out a major role for cell-death-triggering events in AA-mediated actin reorganization.

AA also strongly inhibited macropinocytosis (Figs. 9C and D), a process known to be dependent on actin filament organization 65. Macropinocytosis is functionally similar to phagocytosis, suggesting that the observed effect of AA may be relevant in the context of the tumor microenvironment, since the inability to phagocytize tumor cells is a characteristic trait of TAMs 94. Apart from the G protein regulators alluded to above, another interesting protein in this context is HSP27, which has been described as a regulator of actin filaments and macropinocytosis 54 and is phosphorylated in response to AA at two different sites (Fig. 2C).

### AA signaling regulates extracellular vesicle release

Another intriguing AA-regulated mechanism suggested by GO term enrichment analysis of our phosphoproteomics data is “vesicle-mediated transport” (Fig. 1; Table S7). The 51 proteins linked this GO term (Table S8) encompass G-protein modulators and actin filament modulators (e.g., ARFGEFs, ARHGAP Rho GTPase-activating proteins, RAB family regulators; LIMK1, MAPK1/3 and STK10 protein kinases) as wells as many numerous regulators of membrane trafficking and secretion, including exocyst complex component 7, intersectin-2, the SNARE component SNAP29, synaptogyrin-2 and vacuolar protein sorting-associated proteins, consistent with the established role of Rho family GTPases in vesicle trafficking 95.

EVs comprise a heterogeneous population of membrane vesicles of different cellular origins. Their size varies and encompasses two main classes: exosomes (50-150 nm), which are of endosomal origin, and microvesicles (50-500 nm), which directly bud from the plasma membrane 69. Their overlapping size spectrum and similar compositions, hamper their distinction after isolation from culture supernatants.

In the present study, we were able to confirm the prediction of the GO term enrichment analysis experimentally by demonstrating an increased release of particles upon AA treatment within a size range expected for EVs (Fig. 10A and B). These particles were sensitive to detergent (Fig. 10C) confirming their lipid nature and were endowed with membrane organizers, i.e., the tetraspanins CD9, CD63 and CD81 (Fig. 10D). These tetraspanins are classical exosomal markers 70, indicating that AA induces the release of exosome-like vesicles from MDMs. In agreement with these results, it has been reported by others that M2-like polarization of human macrophages increases the release of EVs 96. Interestingly, our data also showed that AA treatment led to a higher proportion of tetraspanin-positive particles relative to solvent controls (Fig. 10D). While CD9 and CD81 have been associated with both exosomes and microvesicles, CD63 has been shown to be enriched in exosome populations in different experimental systems 69. Taken together, these data suggest that AA boosts the release of EVs, and in particular exosome-like particles.

As discussed above, our data demonstrate that AA treatment triggers actin-filament rearrangement and Ca^2+^ release in MDMs. Interestingly, both events have been previously linked to the EV releasing process 69, lending further support to our conclusions. In view of the prominent role of EVs in the microenvironment of OC 97-100, it is tantalizing to speculate that AA might promote tumor progression via the increased release of vesicles carrying pro-tumorigenic and/or immune suppressive proteins, lipids and/or nucleic acids from macrophages. In line with this hypothesis are the previously reported tumor tropism of macrophage-derived EVs 101 and the tumor-promoting role of EVs in cancer 6. It will therefore be of great interest to determine the cargo of these vesicles by a combined omics approach in future studies.

## Theranostic implications

Our work has uncovered two levels within the AA-governed signaling network that are associated with the clinical outcome of AA and may thus provide prognostic and/or therapeutic options. The first level is extracellular AA itself. As previously shown, the ascites concentration of AA associated with a short RFS 13. AA is generated either intracellularly or extracellularly from phospholipids by cytosolic or secretory PLA2 isoforms (cPLA2 and sPLA2), constituting a family of 21 proteins 72, 74. As reported in the present study, *PLA2G2A* showed the highest variance of expression in tumor tissue from different patients and reached the highest expression level among all *PLA2* isoforms in a subset of patients. Intriguingly, the latter patients are characterized by a short OS and a short RFS, while no association with a short OS was observed for others *PLA2* isoforms. sPLA2s are well known as proinflammatory enzymes but their role in human cancer is controversial 73, which may also be reflected by the observation that three *PLA2* isoforms (*PLA2D, PL4a, PLA12D*) are linked to a longer OS of OC (Table 1). Our data suggest that pharmacological inhibition of PLA2G2A might be therapeutically beneficial in OC, although direct experimental evidence is lacking at this point. Furthermore, PLA2G2A might be a useful prognostic marker, as suggested for breast cancer 102, a question to be addressed in future biomarker studies.

We have previously reported that a high ascites levels of PLA2G7 protein is associated with a short RFS 13, which is not the case for *PLA2G7* RNA levels in solid tumor tissue (Table 1). A possible explanation for this apparent discrepancy is the high expression of *PLA2G7* in ascites-associated macrophages 13, which in turn represent the major population of ascites cells in the majority of OC patients, thereby increasing the potential functional relevance of PLA2G7. This would suggest that both tumor-associated PLA2G2A as well as ascites-associated PLA2G7 may be useful theranostic candidates.

The second level is represented by the signal transduction pathways triggered by AA. In this context, signaling components at the crossroads with other pro-tumorigenic pathways are of particular interest. These include CAMKII 103, ASK1 75, p38 104, HSP27 105 and Rho GTPase regulators 106. Modulating the activity of any of these signaling molecules might not only dampen the pro-tumorigenic effect of TAMs but might also reduce the growth, survival and metastatic potential of cancer cells. It is possible that these signal molecules also have prognostic value in OC, but this option can only be assessed once data directly addressing this issue are available.

## Figures and Tables

**Table 1:**
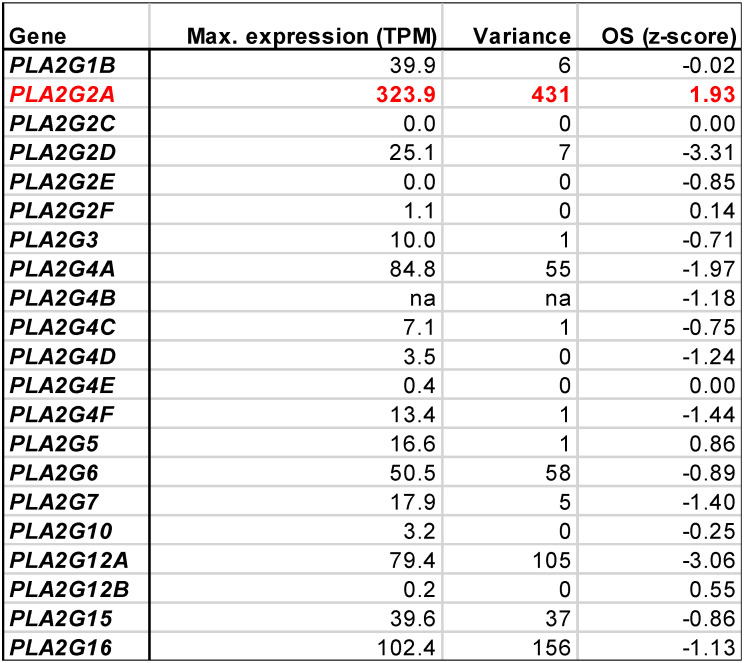
Expression of phospholipase A2 genes in in solid tumor tissue (TCGA RNA-Seq data) and association of expression with overall survival (OS, PRECOG data). A positive z-score indicates a hazard ratio > 1, a negative z-score a hazard ratio < 1; |z| = 1.96 corresponds to *p* = 0.05. The expression data show the maximum expression levels (TPM) and the variance of expression among samples (variance is the average squared deviation from the mean). na: not available
