# Supplementary material for: Phosphoproteomics identify arachidonic-acid-regulated signal transduction pathways modulating macrophage functions with implications for ovarian cancer
Source: Theranostics. 2021 Jan 1;11(3):1377–95. doi: 10.7150/thno.52442 (PMC7738879; doi:10.7150/thno.52442)
Supplement: Supplementary file 1 — Supplementary figures. [file thnov11p1377s1.pdf]

Phosphoproteomics identify arachidonic-acid-regulated signal transduction pathways modulating macrophage functions in ovarian cancer

SUPPLEMENTAL FIGURES

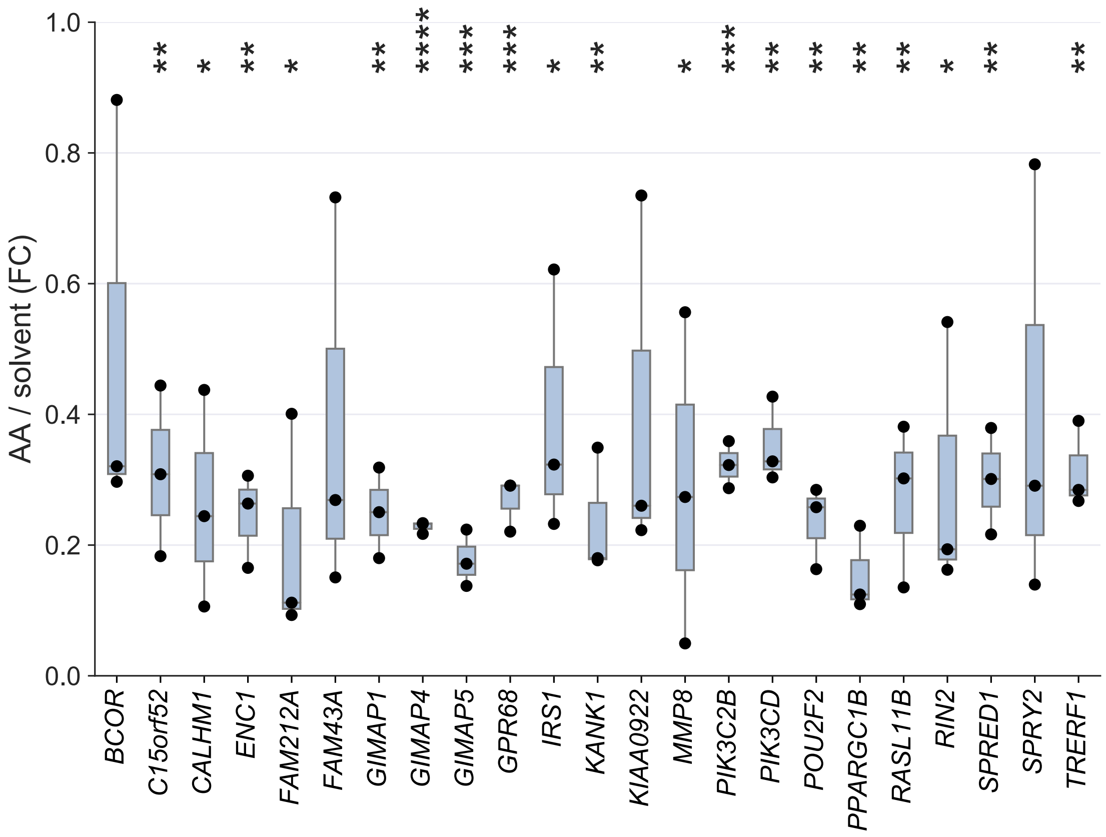

Figure S1. RNA-Seq results for the top AA-repressed genes (FC < 0.33; TPM > 3 in solvent-treated cells).

Box pots show medians (line), upper and lower quartiles (box) and range (whiskers). Significance tested by t test: **** p < 0.0001; *** p < 0.001; ** p < 0.01; * p < 0.05 for AA versus solvent (p values adjusted for multiple hypothesis testing by Benjamini Hochberg correction).

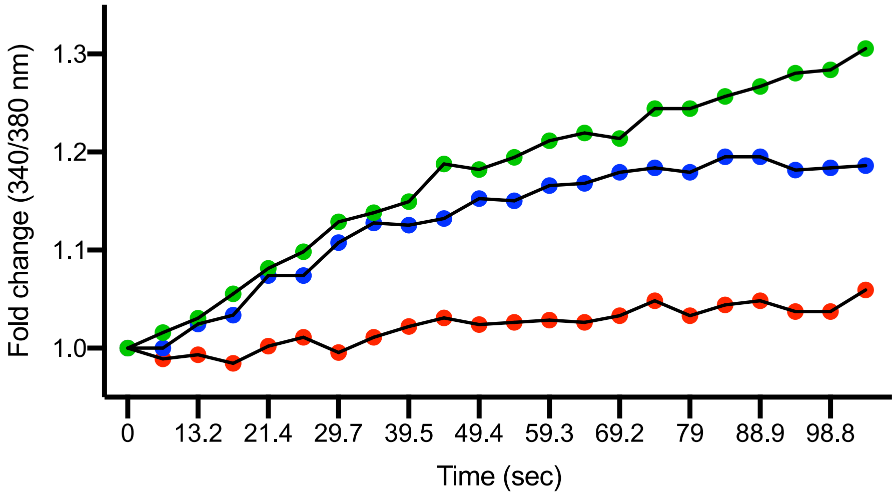

Figure S2. Induction of intracellular free Ca2+ levels by AA and ETYA.

MDMs were treated with 50 µM AA (green), ETYA (blue) or solvent (red) and analyzed as described in Materials and Methods. Results are represented as the increase in fluorescence at 340 nm to 380 nm over time after addition of AA, ETYA or solvent. Values were normalized to 1 for the first data point.

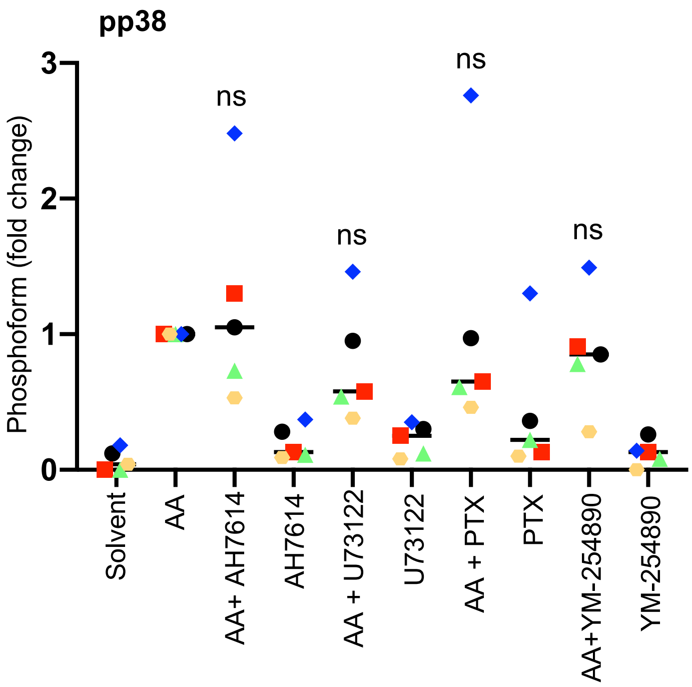

Figure S3. Donor-dependent effect of FFAR4, PLCγ and GPCR inhibitors on AA-induced p38 phosphorylation.

MDMs were treated with AA in the presence of the FFAR4 antagonist AH7614, the PLCγ inhibitor U-73122, the $G_{i/0}$ inhibitor pertussis toxin (PTX), the $G_{q/11}$ inhibitor YM-254890 or solvent. The cells were pretreated either with 50 µM AH7614, 10 µM U73122, 5 µM YM-254890 for 30 min or 1 µg/ml PTX for 3 h prior to treatment with 50 µM AA. p38 and pp38 were visualized by immunoblotting. Plot showing the quantification for n = 5 biological replicates. Data points represents individual samples, horizontal lines indicate the median. Values were normalized to samples with AA only. Statistical analysis was performed by paired t test against samples treated with AA only. ns: not significant.

A

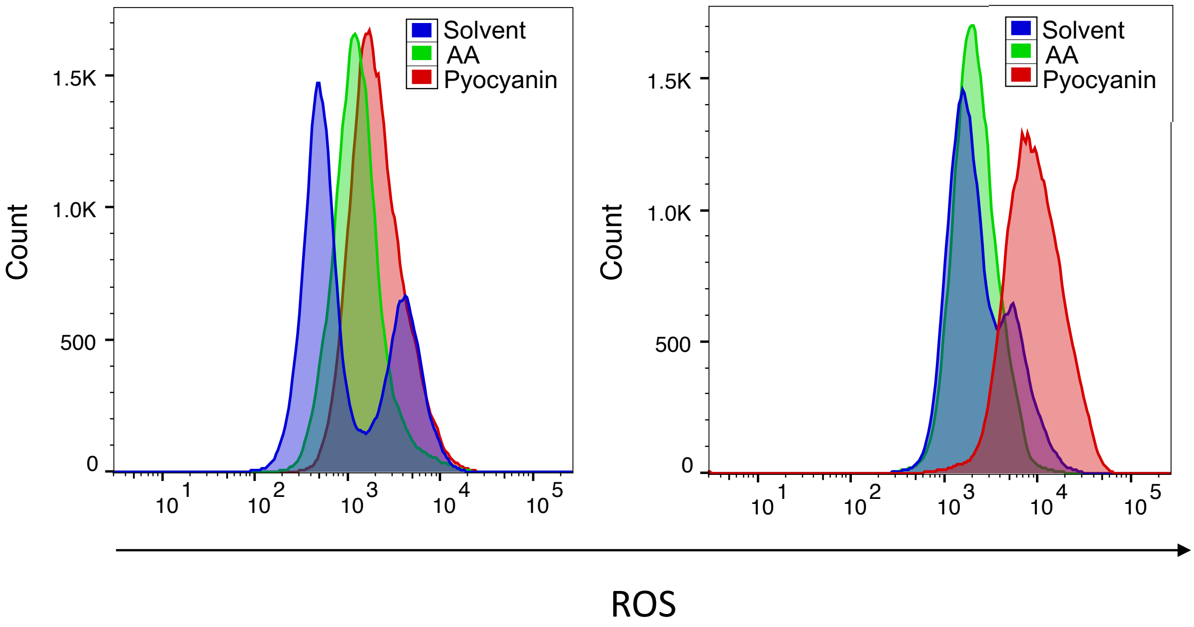

B

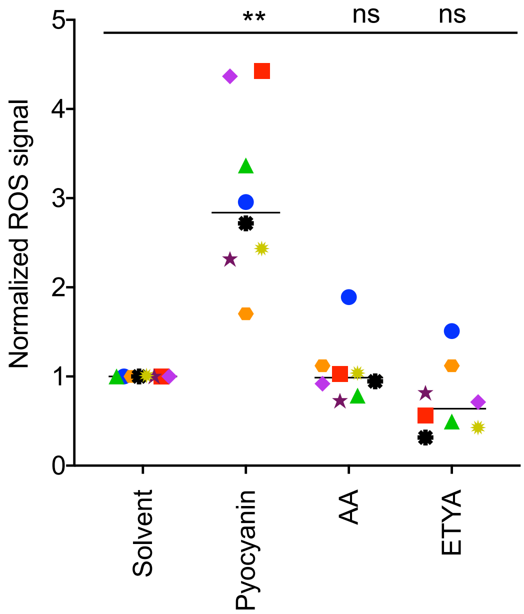

Figure S4. Induction of ROS production by AA on the AA-mediated phosphorylation of p38.

(A) Flow cytometric analysis of AA-induced total ROS (left plot) and superoxide (right plot) generation in MDMs. The cells were either treated with 50 µM AA or with 200 µM pyocyanin to induce oxidative stress as a positive control for 30 min. The histograms depict the data for one donor showing ROS induction by AA and pyocyanin.

(B) Evaluation of 8 biological replicates analyzed as in panel A. The data reveal a strong donor-dependence of ROS induction by AA, but not by pyocyanin. Data points represent individual samples, horizontal lines indicate the median. **$p < 0.01$ by t test; ns, not significant.

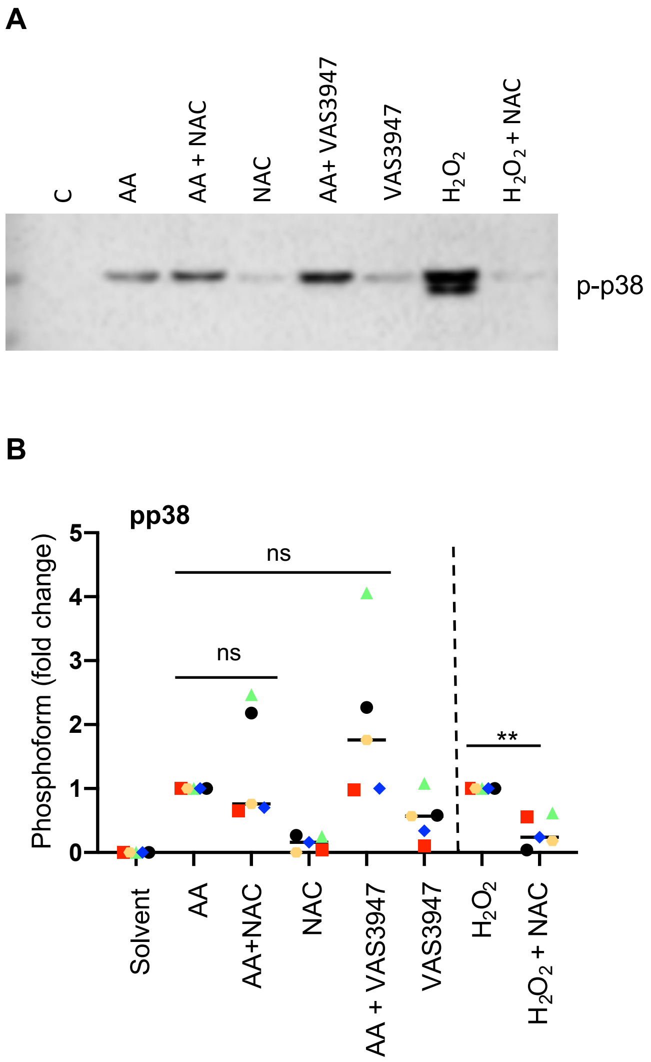

Figure S5. Donor-dependent effect of ROS quenching on the AA/ETYA-mediated phosphorylation of p38. MDMs were incubated with 50 µM of AA for 30 minutes in the presence of N-acetylcysteine (NAC) or the NOX inhibitor VAS3947.

(A) Example of the ROS-independent induction of p38 by AA. The cells were pretreated with 5 mM NAC for 90 min or 2 µM VAS3947 for 60 min prior to treatment with 50 µM AA or 2 mM H_2O_2 for 30 min. H_2O_2 is used as positive controls to validate the efficacy of NAC. For other examples showing the donor-dependent effect of ROS quenching on the AA/ETYA-mediated phosphorylation of p38 see Fig. S5.

(B) Quantification of n = 5 biological replicates as in panel A. Data points represents individual samples, horizontal lines indicate the median. Statistical analysis was performed by paired t test: ** $p < 0.01$ against samples treated with AA only (left from dashed line). ns: not significant.

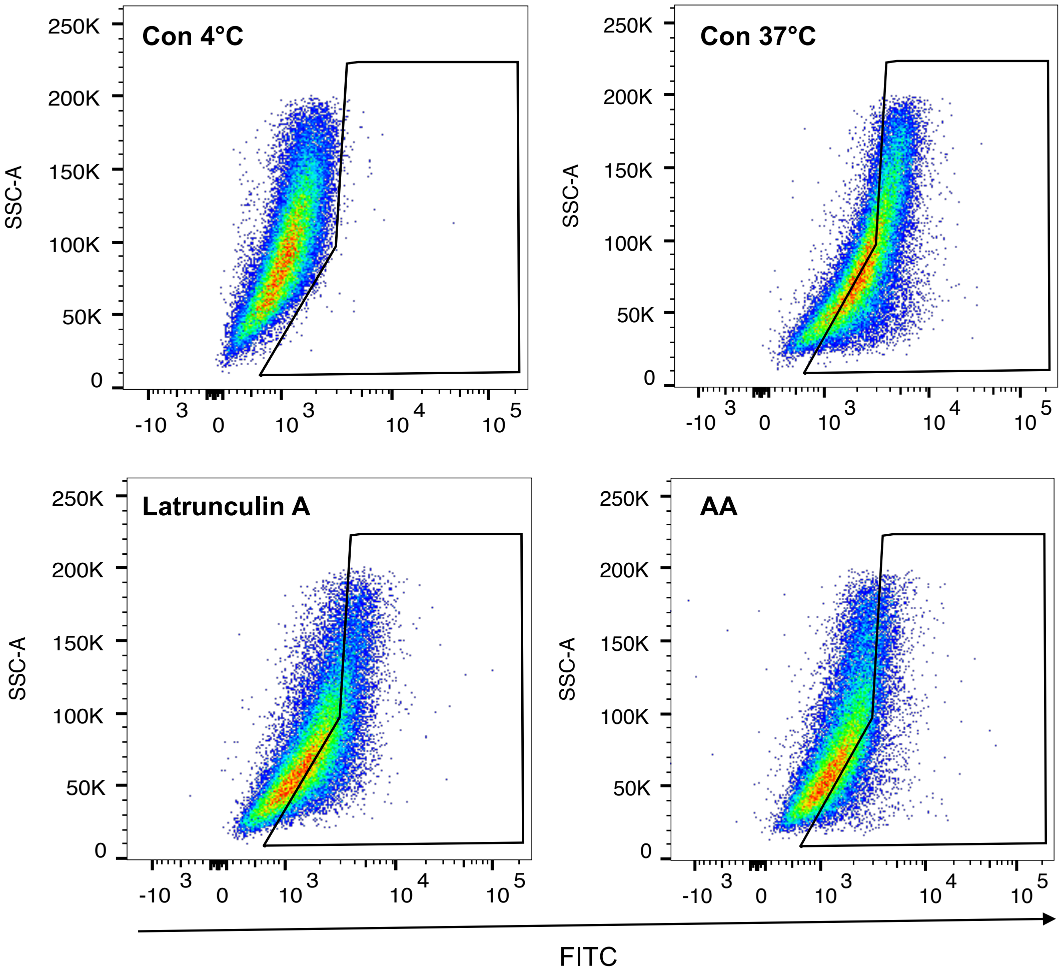

Figure S6. Gating strategy for the flow cytometric analysis in Fig. 9C and D.

For details see legend to Fig. 9.

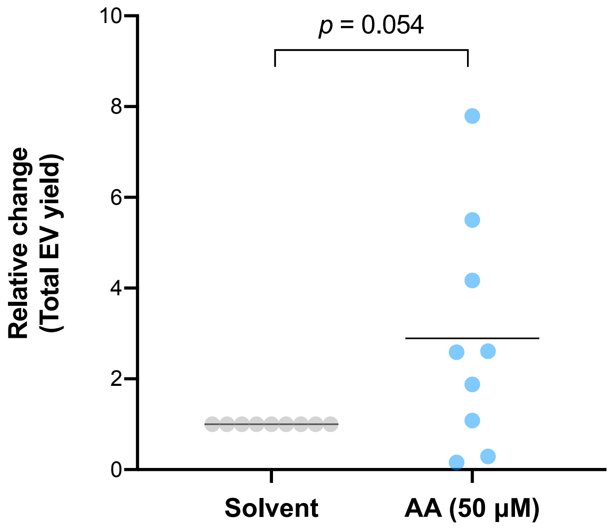

Figure S7. Quantification of EVs isolated by differential ultracentrifugation from conditioned media of AA- and solvent-treated MDMs.

Particles were isolated from conditioned media after 24 h of treatment with AA (blue dots) or solvent (grey dots) through differential centrifugation as detailed in Materials and Methods (12 ml of media from a 150 mm petri dish were suspended in a final volume of 50 µl of PBS). The relative change of total MDM-derived EVs preparations was determined by HSFC. Values were normalized to 1 for EV samples from solvent-treated cells. Data show results from three independent isolation experiments (n = 9 donors). Significance was tested by paired t test (AA versus solvent).

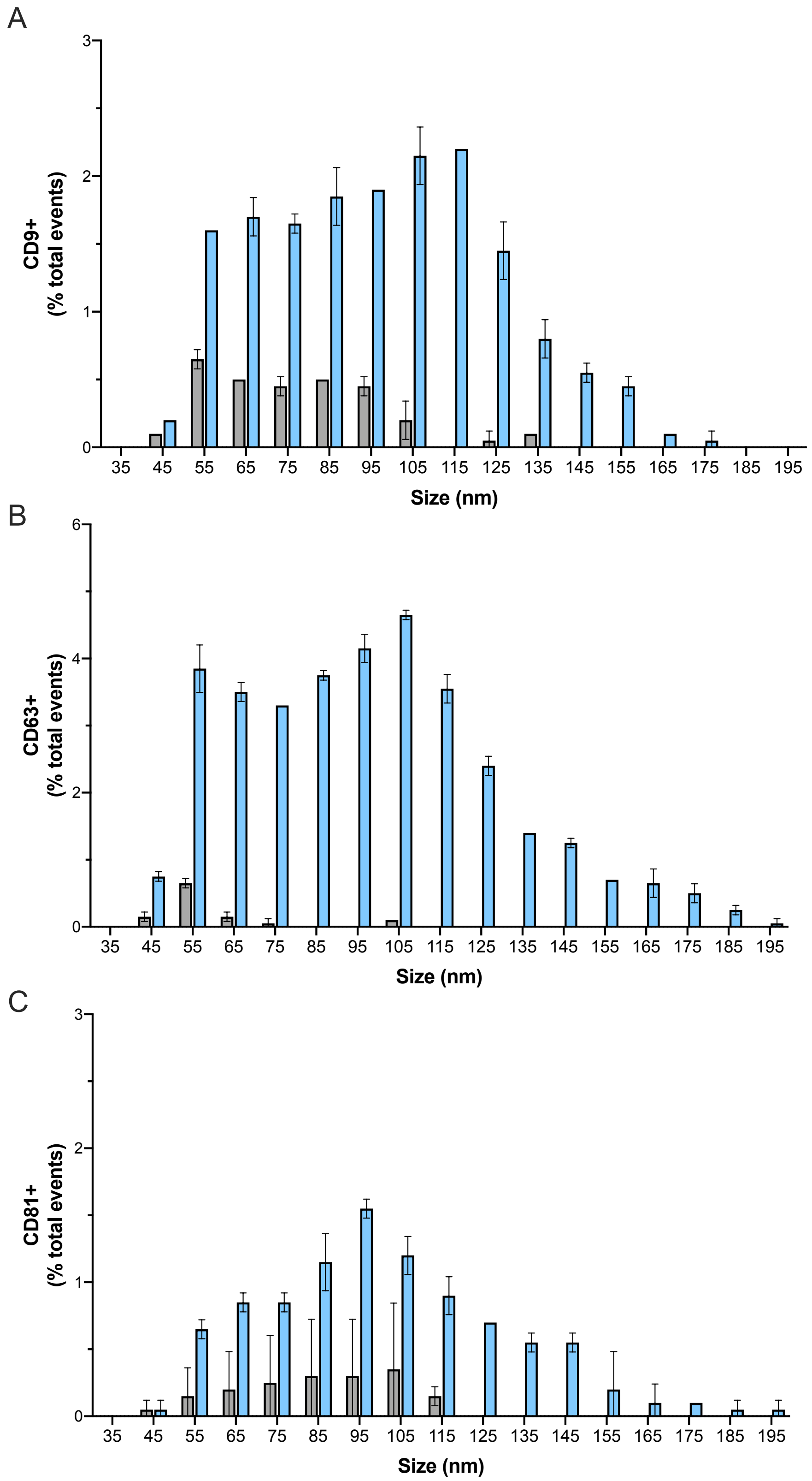

Figure S8. Histograms of immunostained particles analyzed in Fig. 10D.

The percentage of positive events was plotted against particle size with a bin width of 10 nm for AA (blue bars) and solvent control (grey bars) samples for a representative donor. Data show mean (± SD) from two technical replicates of CD9 (A), CD63 (B) and CD81 (C) positive events normalized to the total number of particles acquired by HSFC.

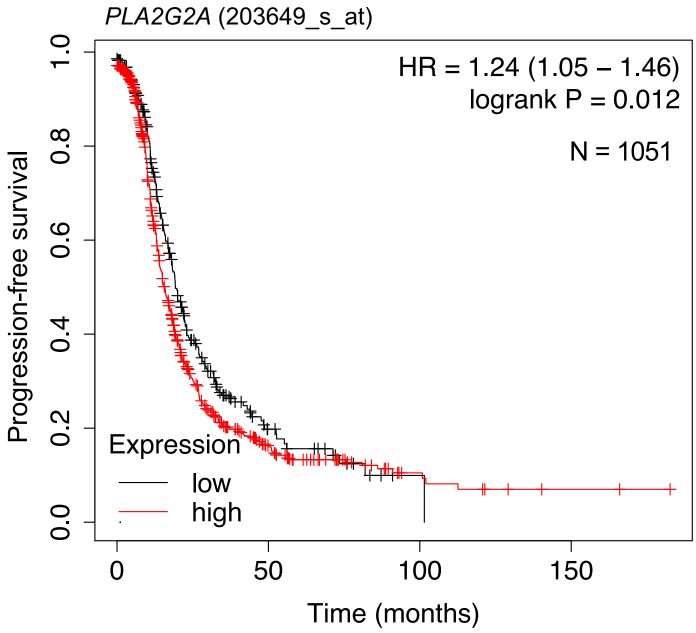

Figure S9. Association of *PLA2G2A* RNA expression with a short progression-free survival of OC.

The plot was constructed by Kaplan Meier Plotter (2017 version; http://kmplot.com).
